# Route optimization for urban hazardous materials transportation under time-varying road networks

**DOI:** 10.1371/journal.pone.0361762

**Published:** 2026-11-03

**Authors:** Hechen Li

**Affiliations:** School of Mechanical Engineering, Xi’an Technological University, Xi’an, Shaanxi Province, China; Indian Institute of Technology Guwahati, INDIA

## Abstract

This paper investigates a time-dependent urban hazardous materials (hazmat) transportation routing optimization problem, where vehicles depart from a distribution center to serve a set of customers. The decision variables include both the travel path and the departure time at each node, with the option for vehicles to wait if necessary. The objective is to minimize both the total transportation risk and cost, which consists of travel costs and overtime penalty costs. To address this, we propose PPO-MOEA/D, a reinforcement-learning-based approximation algorithm that enhances the performance of the conventional MOEA/D. To reduce computational complexity, we introduce a preprocessing strategy for departure-time decision identification and optimization, focusing only on critical nodes. Proximal Policy Optimization (PPO) is utilized to dynamically generate high-quality initial solutions and adaptively select crossover and mutation operators, improving evolutionary efficiency. Extensive experiments on instances with varying scales demonstrate that optimizing departure times significantly reduces transportation risk. Compared to the traditional MOEA/D, PPO-MOEA/D shows superior performance in terms of convergence speed, solution quality, and computational efficiency. Additionally, we analyze the trade-off between computational cost and solution quality across different population sizes and investigate how total transportation risk varies across time periods.

## Introduction

With the rapid advancement of globalization and industrialization, hazardous materials transportation has become increasingly indispensable for ensuring the stable operation of diverse industrial supply chains. In general, hazardous materials are defined as substances that may pose substantial threats to public health, property safety, and the surrounding environment under specific conditions, encompassing a wide spectrum of commodities such as chemical raw materials, fuels, and industrial chemicals. These materials constitute fundamental inputs for major pillar industries, including chemical engineering, petroleum processing, and energy production, thereby underpinning modern socioeconomic development. Recent statistics indicate that the global hazmat transportation market is projected to reach USD 212.8 billion [1]. In particular, the Chinese market has expanded to over CNY 2 trillion, supported by approximately 13,000 hazmat logistics enterprises, 1.62 million employees, and 575,000 dedicated transport vehicles [2]. Nevertheless, the continuous growth in transportation scale is accompanied by a corresponding escalation in potential risks. Once an accident occurs during hazmat transportation, it frequently induces severe secondary consequences, such as leakage, fire and explosion events, and toxic exposure, which may further amplify human casualties and environmental damage on a broader spatial scale. Therefore, in the development of transportation plans, decision-makers are required not only to improve operational efficiency and reduce logistics costs, but also to achieve robust risk mitigation and effective safety control.

In the context of time-dependent road-network-based optimization for hazardous materials (hazmat) transportation, both link travel times and en-route risk exposure exhibit pronounced temporal variability, particularly in urban environments. Accordingly, prior studies addressing time-dependent hazmat routing can be systematically classified into three representative modeling paradigms. A common approach is to describe travel times using piecewise-defined functions, where the planning horizon (typically one day) is divided into multiple discrete time intervals, and each road segment is assigned interval-specific travel-time parameters (e.g., piecewise-constant or piecewise-linear travel-time profiles). Such formulations strike a balance between realism and tractability, as they capture congestion disparities across time periods while preserving computational feasibility. They are therefore widely employed to explicitly couple route decisions with arrival-time evolution through discrete-time representations or scheduling-oriented constraints [3]. Another stream models link travel time as a continuous function of the vehicle’s entry time into a road segment, often derived from speed-time relations or congestion evolution functions. This continuous representation better reflects the smooth temporal dynamics of traffic conditions; nevertheless, it inevitably leads to nonlinear time-propagation (recursive) relationships along a route. As a result, exact optimization is typically challenging, and solution procedures often rely on discretization-based approximations or heuristic/metaheuristic algorithms to ensure computational practicality [4]. Beyond travel-time variability, a third line of research emphasizes that time dependence may also stem from changes in road accessibility or capacity, such as time-window-based traffic restrictions, temporary road closures, or time-dependent capacity constraints. Under such conditions, certain links may become infeasible during specific periods, or vehicle movements may be subject to additional operational limitations (e.g., restrictions associated with permissible loads or admissible traffic volumes), thereby further complicating feasible route construction [5].

Notably, in most existing studies, a vehicle’s temporal schedule is largely endogenously determined by the visiting sequence and the resulting travel times, while explicit departure-time decisions at intermediate nodes, particularly those that allow intentional waiting, are seldom incorporated as optimization variables. As a consequence, the potential of actively shifting departure times to enter downstream links at more favorable moments (e.g., to alleviate congestion impacts and thereby reduce risk exposure) remains insufficiently explored. Therefore, although travel times and risks may both vary in practice, the specific problem of jointly optimizing vehicle routes and node departure times to minimize total transportation risk and cost, with a focus on time-dependent risk parameters, still calls for systematic investigation. Motivated by this practical context and the identified research gap, this paper formulates and studies a time-dependent urban hazmat vehicle routing optimization problem, with the main contributions summarized as follows:

(1) For the proposed time-dependent urban hazmat vehicle routing optimization problem, we formulate a bi-objective mixed-integer programming (MIP) model. Distinct from most existing studies, the proposed risk representation explicitly integrates accident probability, vehicle load (i.e., the quantity of hazardous materials involved), and the exposed population affected by an accident. In particular, the population along each road segment is allowed to vary across time periods, thereby capturing time-of-day heterogeneity in risk exposure. Transportation risk is quantified as the product of accident rate and accident consequence, and the model jointly optimizes vehicle departure-time decisions with the objectives of minimizing the total transportation risk and the overall transportation cost, which comprises travel cost and overtime penalty cost.
(2) To address the computational challenges induced by time dependency and multi-objective decision-making, we develop a deep reinforcement learning-based approximation framework, termed PPO-MOEA/D, which is tailored to the structural characteristics of the problem. Three key enhancements are introduced to improve algorithmic performance: A departure-time decision identification and optimization preprocessing procedure is proposed to filter out departure-time decisions at non-critical nodes, thereby reducing computational burden and search complexity; A PPO-trained agent is employed to dynamically construct high-quality initial solutions for MOEA/D, improving the effectiveness of subsequent evolutionary search; Another PPO-trained policy is designed to adaptively select crossover and mutation operators according to the characteristics of different evolutionary stages, further accelerating convergence and enhancing the evolutionary efficiency of MOEA/D.
(3) We conduct extensive computational experiments to examine the trade-offs between computational efficiency and solution quality under different population sizes, and to investigate how time-period-dependent risk variations influence optimal routing decisions. The results indicate that temporal risk fluctuations can substantially compress the feasible search space, which necessitates adaptive waiting strategies. Specifically, when risk levels increase over time, it is preferable to reduce waiting time to avoid entering higher-risk periods; conversely, when risk levels decrease, extending the waiting window may yield improved solutions. Moreover, certain time-period risk changes exhibit a buffering effect, under which medium-scale networks demonstrate the strongest adaptability. From a managerial perspective, these findings suggest that refined time-period control combined with waiting strategies can effectively bypass high-risk intervals, and that large-scale networks may be decomposed into moderately sized subnetworks to enhance practical implementability.

The remainder of this paper is organized as follows. Section 2 presents a comprehensive review of the related literature, with particular emphasis on three major research streams: the hazardous materials vehicle routing problem (HMVRP), the time-dependent vehicle routing problem (TD-VRP), and the time-dependent hazardous materials vehicle routing problem (TD-HMVRP). Section 3 provides a detailed problem description and formulates a time-dependent urban hazmat vehicle routing optimization model. Section 4 introduces the proposed solution methodology, namely the improved MOEA/D framework (PPO-MOEA/D). Section 5 evaluates the effectiveness of the proposed model and algorithm through extensive computational experiments, and further conducts a sensitivity analysis to derive managerial insights. Finally, Section 6 concludes the paper and outlines directions for future research. The problem studied in this paper is structurally a variant of the classical Vehicle Routing Problem (VRP), which was first introduced by Dantzig and Ramser [6] and extensively reviewed by Laporte [7]; the VRP in turn generalizes the Traveling Salesman Problem (TSP), a fundamental combinatorial optimization problem [8]. When travel times vary with the departure time, the problem becomes a time-dependent variant of the VRP/TSP, in the context of dynamic traffic conditions [9, 10]. Specifically, when the fleet size reduces to one and the risk objective is disregarded, our formulation collapses to a time-dependent TSP with node waiting decisions; If the number of vehicles is greater than one, it becomes a hazmat VRP under time-dependent conditions. Accordingly, the proposed model inherits the fundamental combinatorial structure of VRP/TSP, and the standard VRP modeling requirements, including route continuity, flow conservation, and subtour elimination, must be explicitly enforced. The complete mathematical formulation incorporating these elements is presented in Section 3.

## Literature review

This study focuses on a time-dependent road-network-based vehicle routing optimization problem for hazardous materials transportation. Accordingly, this section reviews the relevant literature from three complementary perspectives: Hazmat routing optimization under static road networks with deterministic travel times, vehicle routing optimization in time-dependent networks, and hazmat vehicle routing optimization under time-varying road networks. Finally, we summarize the similarities and differences between existing studies and the proposed research, and highlight the specific research gap that this paper aims to address.

### Hazmat routing optimization under static road networks with deterministic travel times

Hazardous materials vehicle routing optimization can be viewed as a specialized extension of the classical vehicle routing problem (VRP). Unlike conventional freight distribution, hazmat transportation involves inherently dangerous cargos, which makes risk a critical factor that must be explicitly incorporated into decision-making. In what follows, we review the related studies on multi-objective hazmat vehicle routing optimization, with particular attention to formulations that jointly consider operational efficiency and transportation safety.

Based on practical considerations, a growing body of research has incorporated a variety of risk quantification schemes into hazmat routing optimization. For instance, Zhang et al. accounted for disaster avoidance and near-failure conditions, defining risk as a function of vehicle load and formulating a bi-objective model that minimizes both the maximum risk and the transportation cost, which was solved using the ε-constraint method and a fast approximation algorithm [11]. In a subsequent study, Zhang et al. further distinguished multiple risk categories, including transportation risk, storage risk, and transshipment-node risk, and developed a medical waste collection routing model with the objectives of minimizing total cost and maximum risk, which was tackled via a global optimization approach and a two-stage solution procedure [12]. Similarly, Wang et al. emphasized that medical waste collection must explicitly consider infectious risk, coordination among multiple disposal facilities, and disaster-avoidance strategies. They proposed a bi-objective routing model that minimizes the maximum infection risk and transportation cost, constructed under a two-commodity modeling framework, and solved it using a weighted ε-constraint method combined with a fast approximation algorithm [13]. Ma & Li investigated the impact of time-varying hazardous waste weight on transportation risk, establishing a multi-period waste collection model that simultaneously minimizes transportation cost, transportation risk, and the inter-period equity of risk distribution. Their formulation allows incomplete or delayed collection and was solved using CPLEX [14]. In addition, Ghannadpour et al. proposed a composite risk metric that integrates arrival time, waste quantity, infection probability, and hazard intensity, and developed a medical waste collection-and-transport model with the objectives of minimizing risk, fuel consumption, and transportation cost, which was solved using an adaptive multi-objective evolutionary algorithm (MOSAEA) [15]. Chai et al. introduced a driving-risk-aware evaluation scheme and formulated a hazmat routing model aiming to minimize both risk and cost, which was addressed using a non-dominated genetic algorithm [16]. Taslimi et al. developed a periodic, load-dependent capacitated vehicle routing model with objectives of minimizing transportation and occupational risks as well as transportation cost, and solved it through a decomposition-based heuristic approach [17]. Moreover, Hu et al. adopted an integrated risk representation by balancing the weights of the ordered weighted averaging (OWA) risk model and the state variable weight (SVW) risk model. They formulated a multi-depot vehicle routing model to minimize total risk and proposed a constraint-reduction mechanism together with a hybrid parallel genetic algorithm embedded with variable neighborhood search [18]. Tao et al. quantified accident probability via a safety assessment (PSA) model and evaluated radiological consequences using a PEHR composite severity index incorporating vulnerability and resilience, thereby constructing a routing model for radioactive materials that minimizes radiological risk cost, time cost, and economic cost; the optimal route was identified using a grey relational analysis (GRA) model [19]. Finally, Zhang et al. integrated exposed population, travel time, and emergency response effectiveness into a sustainability-oriented risk measure, and proposed a bi-level programming model that jointly optimizes route planning and emergency response center location decisions, with the objectives of minimizing exposed population, transportation time, and uncovered transportation demand [20].

Several studies have further extended hazmat routing models by incorporating special operational conditions that arise in real-world transportation practice. For example, Wang et al. developed a multi-depot heterogeneous vehicle routing model that explicitly accounts for vehicle fleet diversity, with the objective of minimizing vehicle activation costs, overtime expenses, and risk-conversion costs. To solve the resulting model, they proposed a bidirectional tuning heuristic algorithm and a particle swarm optimization (PSO) approach [21]. Focusing on convoy-avoidance requirements in hazmat transportation, Wang et al. formulated a bi-objective vehicle routing model that simultaneously minimizes the maximum risk and the transportation cost, where the model explicitly discourages vehicles from traveling in platoons; the ε-constraint method was employed to obtain Pareto-optimal solutions [22]. More recently, Zhai et al. proposed an explosion-risk-oriented route selection and planning framework, aiming to minimize both explosion risk and accident consequences. Their work established a multi-level quantitative risk assessment model that integrates traffic speed, vehicle status, accident probability, and damage severity, and adopted a depth-first search algorithm to derive the optimal transportation route [23].

Beyond the aforementioned studies, a number of researchers have explicitly incorporated uncertainty into hazmat transportation modeling. In practice, transportation environments are inherently complex due to the intertwined effects of traffic conditions, meteorological factors, and human-related disruptions, which may induce uncertainty in key elements such as risk exposure, travel time, and customer demand. Such uncertainty complicates the quantification of both risk and cost, and can substantially alter route-selection decisions. For instance, Shen et al. considered uncertain vehicle speeds and dynamically evolving customer demands, and formulated a multi-trip medical waste collection vehicle routing problem with multiple transfer depots, aiming to minimize both total cost and infection risk. They developed a two-stage solution framework that combines a knowledge-guided interval multi-objective shuffled frog leaping algorithm with a neighborhood search procedure [24]. Xie & Cao investigated a multi-modal and multi-route hazmat transportation problem under uncertainty, proposing a mathematical model that minimizes transportation risk and transportation cost, which was solved using a genetic algorithm (GA) [25]. Furthermore, Sirbiladze et al. addressed uncertainty in both vehicle travel time and road capacity, and constructed a fuzzy vehicle routing model for extreme conditions with the objective of minimizing decision risk; the model was solved via a dancing links algorithm inspired by D. Knuth [26]. In a related vein, Hu et al. focused on incorporating dynamic traffic conditions to better capture real-world fluctuations, and developed a hazmat routing model with the objectives of minimizing transportation cost and risk, which was solved using a GA-based approach [27]. Finally, Wang & Liang leveraged trajectory data from hazmat transportation vehicles to establish a routing model that minimizes total cost and risk, proposed a high-risk route avoidance principle, and designed a hybrid solution method integrating an improved depth-first search (DFS) algorithm with a fast non-dominated sorting procedure [28]. The content is summarized in Table 1 as shown.

**Table 1:** A Summary of hazmat vehicle routing optimization studies under static road networks with deterministic travel times.

Reference	Objective		Special considerations	Solution method
	Risk	Cost		
Wang et al (2017)	√	√	Avoiding echelon travel	Exact methods
Taslimi et al (2020)	√	√	Load-dependent risk; Occupational risk	Heuristic methods
Wang et al (2020)		√	Multi-depot heterogeneous vehicle	Heuristic methods
Xie & Cao (2020)	√	√	Multi-modal; Multi-route; Uncertainty	Heuristic methods
Wang & Liang (2020)	√	√	Real-time data; High-risk avoidance	Metaheuristic Algorithm
Zhang et al (2021)	√	√	Catastrophe avoidance; Failure edge; Load-dependent risk	Exact methods
Ghannadpour et al (2021)	√	√	Arrival time, waste volume, infection probability, and hazard intensity-based risk	Heuristic methods
Tao et al (2022)	√	√	Safety assessment-based accident probability and radiation consequence evaluation	Data-driven Methods
Sirbiladze et al (2022)	√		Fuzzy uncertainty in vehicle time and road capacity	Exact Algorithm
Hu et al (2022)	√	√	Dynamic traffic adaptation	Heuristic methods
Zhang et al (2022)	√		Exposure population, transportation time, and emergency response efficiency-based risk; Bilevel programming model	Heuristic methods
Wang et al (2023)	√	√	Multiple disposal centers; Disaster avoidance	Exact Algorithm
Ma & Li (2023)	√	√	Time-varying hazardous waste weight; Incomplete or delayed waste collection	Exact Algorithm
Chai et al (2023)	√	√	Driving behavior based risk	Heuristic methods
Hu et al (2023)	√		Multi-depot; OWA^a^ and SVW^b^ weight balance based risk	Heuristic methods
Zhai et al (2024)	√		Explosion accident risk	Exact Algorithm
Zhang et al (2025)	√	√	Comprehensive risks including storage risk	Exact Algorithm
Shen et al (2025)	√	√	Uncertainty in vehicle speed and dynamic customer demand	Metaheuristic Algorithm

^a^OWA: Ordered Weighted Averaging. ^b^SVW: State Variable Weighting.

### Vehicle routing optimization in time-dependent road networks

In conventional vehicle routing studies, the term “time-dependent road network” typically refers to the phenomenon that link travel times vary with the departure time or the time period, thereby shaping vehicles’ arrival times and ultimately affecting the overall transportation cost. Consequently, this stream of research primarily centers on the temporal dynamics of travel time. Within the domain of the time-dependent vehicle routing problem (TDVRP), a large body of work has focused on optimizing a single operational objective, such as minimizing distribution cost, travel time, or the number of routes. For example, Carić and Fosin utilized historical traffic data to transform time-varying travel times into predictable time-period travel-time profiles and congestion zones. They formulated a model with the objectives of minimizing the number of routes and the total travel time, and solved it by adapting classical VRP algorithms to comply with time-dependent travel-time rules [29]. Khanchehzarrin et al. highlighted the periodicity of traffic patterns and departure-time-related intelligent travel times, proposing a mathematical model that minimizes both fixed and variable costs, which was addressed using a tabu search optimization algorithm. In addition, time dependency has been extensively investigated in the context of electric vehicle routing [30]. Considering uncertainty in dynamic urban traffic networks, Fan introduced a flexible energy-consumption estimation strategy to mitigate range anxiety, and established a multi-depot semi-open time-dependent electric vehicle routing model aimed at minimizing total distribution cost; a two-stage hybrid ant colony optimization algorithm was developed for solution [31]. Similarly, Lu et al. proposed a cost-minimization electric vehicle routing model that explicitly incorporates departure-time decisions and variations in vehicle speed, and solved it using an iterative variable neighborhood search method [32]. Beyond travel time alone, Ilabaca et al. captured the time-varying nature of both travel time and service time in urban delivery operations. They formulated a mathematical model with the objective of minimizing the maximum arrival time among all vehicles and workers, and solved it using a black widow optimization algorithm [33].

In contrast to the single-objective formulations discussed above, a growing number of TDVRP studies have adopted multi-objective optimization frameworks to capture the trade-offs among competing operational criteria. For example, Menares et al. treated the delivery failure probability, service time, and service cost as time-dependent factors, and formulated a time-dependent vehicle routing model that minimizes both operational cost and the delivery failure rate; the model was solved using NSGA-II [34]. Yue et al. integrated time-dependent characteristics into the green vehicle routing problem, developing a multi-objective model that simultaneously considers travel time, carbon emissions, and customer satisfaction, and proposed a two-stage solution framework based on reinforcement learning [35]. Moreover, Zhao et al. employed historical traffic indices to predict time-dependent travel times, and established a TDVRP model that accounts for route heterogeneity under congestion, with the objectives of minimizing total cost and maximizing customer satisfaction. Their solution approach combined a dynamic programming method with a bisection strategy and several multi-objective evolutionary algorithms [36]. In a related study, Poon et al. investigated a time-dependent vehicle routing problem involving both private fleets and public carriers, representing time-dependent travel times via piecewise-linear functions. They formulated a cost-minimization model and developed a hybrid metaheuristic that integrates adaptive large neighborhood search with tabu search [37]. The content is summarized in Table 2 as shown.

**Table 2:** Summary of Studies on the Time-Dependent Vehicle Routing Problem.

Reference	Objective					Special considerations	Solution method
	DFR^a^	CS^b^	CE^c^	MAT^d^	Cost		
Carić & Fosin (2020)					√	Time-varying travel time prediction	Exact methods
Lu et al. (2020)					√	Departure time and vehicle speed variation	Metaheuristic Algorithm
Khanchehzarrin et al (2021)					√	Smart travel time	Metaheuristic Algorithm
Poon et al (2022)					√	Time-dependent with private fleets and public carriers	Metaheuristic Algorithm
Menares et al (2023)					√	Delivery failure probability based cost	Heuristic methods
Zhao et al (2023)		√			√	Time-dependent travel time with congestion prediction	Exact methods; Metaheuristic Algorithm
Fan (2024)					√	Multi-depot semi-open time-dependent	Metaheuristic Algorithm
Ilabaca et al (2025)				√		Time-varying travel and service times in urban delivery	Metaheuristic Algorithm
Yue et al (2024)		√	√		√	Green vehicle routing with time dependency	Data-driven Methods

^a^DFR: Delivery failure rate. ^b^CS: Customer Satisfaction. ^c^MAT: Maximum arrival time. ^d^CE: Carbon emissions.

### Time-dependent hazmat vehicle routing optimization

Building upon the general TDVRP framework, some studies further integrate operational constraints, such as time windows and fleet configuration, with a dual time-varying environment characterized by both travel-time dynamics and risk heterogeneity, thereby yielding formulations that more closely reflect real-world dispatching decisions. Importantly, unlike conventional TDVRP research that primarily emphasizes time-dependent travel times, time dependency in hazardous materials transportation manifests in a broader sense: in addition to temporal variations in link travel time, the level of en-route risk exposure may also vary across time periods due to factors such as population exposure, accident likelihood, and meteorological dispersion conditions (e.g., wind speed).

In the existing literature, one major research stream primarily emphasizes the temporal variability of travel time, by characterizing speed fluctuations across periods, congestion heterogeneity, or time-dependent travel-time functions that explicitly link travel time to the time at which a vehicle enters the corresponding road segment. Within this line of work, Han & Zhu quantified transportation risk using segment-level population exposure and incorporated the time-varying nature of urban road networks through a speed-based time-dependent travel-time function. They formulated a bi-objective routing optimization model that minimizes total transportation risk and total transportation cost, and proposed a hybrid solution approach that combines NSGA-II with simulated annealing to balance global exploration and local refinement, thereby generating Pareto-optimal solutions that better conform to congestion-driven time-of-day patterns [4].

Another research stream places greater emphasis on the time-varying nature of transportation risk itself, arguing that risk assessment is determined not only by the chosen route but also by temporally evolving contextual factors, such as population distribution and meteorological conditions, so that the same road segment may exhibit substantially different risk levels across time periods. For instance, Zandieh and Ghannadpour modeled hazmat transportation risk as a function of material volume, route length, and population density, and formulated a bi-objective optimization problem to minimize transportation risk and travel cost. To capture the temporal impact on population exposure, they incorporated time-dependent population density into the model, employed an interval type-2 fuzzy logic controller to estimate risk, and developed a decomposed type-2 fuzzy multi-objective evolutionary algorithm integrated with adaptive large neighborhood search. This framework enables robust and operationally tractable risk evaluation under time-varying exposure conditions [3]. Furthermore, Liu et al. incorporated time-dependent wind speed into both risk assessment and equity-oriented decision-making, proposing a time-dependent hazmat routing model that jointly minimizes transportation risk and risk equity. Their model was solved using NSGA-II. This line of work highlights that when risk dispersion dynamics and exposed population vary across time periods, focusing solely on minimizing total risk may inadvertently lead to risk concentration, thereby necessitating the integration of equity objectives for more balanced and socially acceptable solutions [38].

Building on these developments, some studies further integrate operational constraints, such as time windows and fleet heterogeneity, into the coupled time-varying risk–travel-time environment, thereby producing models that more closely reflect real-world dispatching requirements. For example, Zhang et al. investigated a multi-depot heterogeneous vehicle routing problem with time windows, where transportation risk is explicitly linked to vehicle load. They formulated a bi-objective optimization model that minimizes total transportation risk and total transportation cost, and proposed a hybrid multi-objective evolutionary approach together with a tailored two-stage solution framework [20]. With a particular focus on heterogeneous fleets, Karouti et al. developed a load- and time-dependent risk metric and constructed a bi-objective mathematical model that simultaneously minimizes total travel distance and transportation risk, which was solved using an improved NSGA-II algorithm [39].

To mitigate the rapid expansion of the search space induced by time dependency and multi-objective decision-making, Chai & He decomposed the hazmat VRP with time windows into a two-stage framework. In the first stage, a Pulse algorithm was employed to precompute a bi-objective Pareto set of candidate paths between the depot and each customer, thereby substantially reducing the solution space. In the second stage, under the joint consideration of time-window constraints and time-varying risk levels, the overall distribution plan was constructed using an ant colony optimization algorithm equipped with customized pheromone updating and state-transition rules [40].

Beyond temporal dynamics alone, key risk-related components in hazmat transportation, such as accident occurrence and leakage events, are inherently uncertain. Consequently, some recent studies have moved from purely time-dependent modeling toward robust decision-making frameworks that jointly accommodate time variability and uncertainty. For instance, Men et al. formulated a hazmat vehicle routing problem with time-window constraints, aiming to minimize both the number of vehicles and the transportation risk. To explicitly capture risk uncertainty, they introduced a robust criterion based on 32 accident scenarios, and developed a hybrid evolutionary algorithm (HEA) for solution [41]. In a related study, Moghaddam & Kianfar considered a bi-objective setting involving travel time and population exposure risk, and represented the unknown accident probability through a fuzzy joint game-theoretic “demon” opponent. Their model integrates route selection with shipment scheduling under time windows, and was solved using a combination of Monte Carlo simulation and fuzzy goal programming [42]. The content is summarized in Table 3 as shown.

**Table 3:** Review of Time-dependent Hazardous Materials Vehicle Routing Problem.

Reference	Objective			Special considerations	Solution method
	Risk	RF^a^	Cost		
Men et al (2020)	√		√	Time window; Robust modeling of transportation risk uncertainty	Metaheuristic Algorithm
Moghaddam & Kianfar (2023)	√		√	Fuzzy game theory-based accident probability	Hybrid Optimization Method
Zandieh & Ghannadpour (2023)	√		√	material volume, route length based risk	Metaheuristic Algorithm
Zhang et al (2023)	√		√	Load dependent; Time-window; Multi-depot heterogeneous vehicles	Metaheuristic Algorithm
Chai & He (2023)	√		√	Time window; Pareto path pruning	Metaheuristic Algorithm
Han & Zhu (2024)	√		√	Population based risk; Time-dependent function based on vehicle speed	Metaheuristic Algorithm
Cui et al (2025)	√		√	Time-dependent risk and capacity management	Metaheuristic Algorithm
Liu et al (2025)	√	√		Time-varying wind speed based risk	Metaheuristic Algorithm
Karouti et al (2025)	√		√	Load dependent; Time dependent	Metaheuristic Algorithm

^a^RF: Risk fairness.

Overall, the above literature review reveals a clear evolutionary trajectory of research on time-dependent hazmat routing optimization, which can be summarized from the three subsections. First, within the domain of hazmat vehicle routing under static road networks with deterministic travel times (HMVRP), the research focus has gradually shifted from the fundamental risk-cost trade-off toward more realistic formulations that incorporate multi-dimensional risk measures and complex operational constraints. In this process, risk assessment has evolved from single-indicator representations to more comprehensive evaluations that integrate multiple risk sources together with scenario-driven constraints. Second, in the field of the time-dependent vehicle routing problem (TDVRP), studies have progressed from single-objective models primarily targeting the minimization of distribution cost or travel time to increasingly rich multi-objective settings that also account for delivery failure rates, customer satisfaction, and carbon emissions. Meanwhile, traffic dynamics have been more explicitly captured through mechanisms such as time-varying speed profiles and congestion prediction. Finally, research on time-dependent hazmat vehicle routing has advanced by jointly considering time-dependent travel times and time-varying exposure environments (e.g., population distribution and meteorological conditions) in risk evaluation and multi-objective decision-making. Moreover, practical elements, including time windows, heterogeneous fleets, fuzzy uncertainty, and risk equity considerations, have been progressively incorporated, thereby driving the research frontier from “risk control on static networks” toward “integrated risk–cost optimization under time-varying road networks.” From a methodological perspective, the majority of existing studies rely on heuristic approaches and multi-objective evolutionary algorithms, which are well suited to handling diverse transportation settings and competing optimization objectives.

Despite the substantial progress achieved along the three aforementioned research streams, studies that jointly model route selection and node departure-time decisions (including intentional waiting) for urban hazmat transportation under time-varying road networks remain relatively scarce, especially when simultaneously optimizing the dual objectives of total transportation risk and total transportation cost. To address this research gap, this paper develops a time-dependent hazmat vehicle routing optimization model that integrates both routing and departure-time decision-making, and further proposes a tailored solution framework, namely PPO-MOEA/D, designed to exploit the structural characteristics of the problem. In addition, a sensitivity analysis is conducted to extract managerial insights, with the ultimate aim of providing practical and actionable decision support for hazardous materials logistics operators.

## Problem description and modeling

### Problem description

Consider an urban distribution network represented by G(V,E), where $V=\{v_{\text{0}},v_{\text{1}},...,v_{n}\}$ denotes the set of nodes and $E=\left\{e\left(v_{i},v_{j}\right)\right|\;v_{i},v_{j}\in V\}$ denotes the set of edges. Specifically, $v_{\text{0}}$ represents the hazmat distribution center (depot), and $v_{\text{1}},v_{\text{2}},...,v_{n}$ denote the set of customer nodes. For any edge $e\left(v_{i},v_{j}\right)$, its length is denoted by $\mu_{ij}$. The entire planning horizon is discretized into D time periods $\left[a^{1},b^{1}),[a^{2},b^{2}),...,[a^{D},b^{D}\right)$. Each period p corresponds to a discrete time interval, during which the transportation risk $r_{ij}^{p}$ associated with edge $e\left(v_{i},v_{j}\right)$ may vary. In other words, $r_{ij}^{p}$ takes different discrete values across different time periods, with the set of possible values denoted by $r_{ij}^{p}\in [r_{ij}^{\text{1}},r_{ij}^{\text{2}},...,r_{ij}^{D}]$. The period to which a vehicle belongs when traversing $e\left(v_{i},v_{j}\right)$ is determined by its departure time from the node $v_{i}$; accordingly, the corresponding risk parameters are selected for that period. Each customer has a demand $q_{i}$ and a delivery time requirement $W_{i}$. The total distribution cost consists of two components: The travel-related operating cost incurred during vehicle movements, and a penalty cost incurred when the actual delivery time exceeds the required deadline. Vehicles are dispatched from the depot to serve all customers and are required to return to the depot upon completion of service. The problem is formulated as a bi-objective optimization model, with the objectives of minimizing the total transportation risk and the total transportation cost.

To further clarify the problem setting, the following assumptions are made:

(1) The distribution center is equipped with K hazmat delivery vehicles, each with an identical maximum capacity of Q.
(2) Each customer is served at most once, and customer demand is non-splitable.
(3) Vehicles are allowed to wait at nodes during the distribution process.
(4) During the traversal of an edge $e\left(v_{i},v_{j}\right)$, the time-vary parameters are assumed to remain constant.

To facilitate subsequent analysis, we define the following decision variables based on the characteristics of the problem. The remaining parameters are summarized in Table 4.

**Table 4:** Definitions of Parameters.

Parameters	Definitions
$\mu_{ij}$	Length of $e\left(v_{i},v_{j}\right)$
$s_{ij}$	Travel speed on $e\left(v_{i},v_{j}\right)$
$t_{ij}$	Travel time required to traverse $e\left(v_{i},v_{j}\right)$
$q_{i}$	Demand of customer $v_{i}$
Q	Maximum capacity of vehicles
$W_{i}$	Delivery time requirement of customer $v_{i}$
$pop_{ij}^{p}$	Exposed population along $e\left(v_{i},v_{j}\right)$ during time period p
$P_{ij}^{p}$	Accident rate on $e\left(v_{i},v_{j}\right)$ during time period p
$c^{\prime }$	Penalty cost per unit time for late delivery
c	Transportation cost per unit distance
λ	Damage coefficient per unit quantity of hazardous materials
K	Fleet size at the distribution center

Binary variable $x_{ijk}$: equals 1 if vehicle k traverses $e\left(v_{i},v_{j}\right)$, and 0 otherwise.

Binary variable $y_{ik}$: equals 1 if vehicle k serves customer $v_{i}$, and 0 otherwise.

Binary variable $z_{ik}^{p}$: equals 1 if the departure of vehicle k from node $v_{i}$ falls within time period p, and 0 otherwise.

Nonnegative variable $u_{ik}$: the remaining load of vehicle k upon arrival at node $v_{i}$.

Nonnegative variable $a_{ij}$: the vehicle load carried on edge $e\left(v_{i},v_{j}\right)$.

Nonnegative variable $A_{ik}$: the arrival time of vehicle k at node $v_{i}$.

Nonnegative variable $d_{ik}$: the departure time of vehicle k from node $v_{i}$.

Nonnegative variable $w_{ik}$: the waiting time of vehicle k at node $v_{i}$.

### Risk metric

The hazardous materials delivery risk is influenced by several factors, including accident rate, vehicle load, and the number of people affected by the accident. Additionally, the accident rate and population along $e\left(v_{i},v_{j}\right)$ vary across different time periods. As a result, the risk in this study is defined as a time-varying risk. To further refine the risk metric, we consider that the risk is a product of the accident consequence and the accident rate. Specifically, the risk associated with $e\left(v_{i},v_{j}\right)$ is given by $r_{ij}^{p}=P_{ij}^{p}C_{ij}^{p}$, where $P_{ij}^{p}$ represents the accident rate on $e\left(v_{i},v_{j}\right)$ during time period p, $C_{ij}^{p}$ represents the consequence of an accident occurring on $e\left(v_{i},v_{j}\right)$ during time period p, which is related to vehicle load and the population exposed. Thus, $C_{ij}^{p}$ can be expressed as $\lambda pop_{ij}^{p}a_{ij}$, where $pop_{ij}^{p}$ is the population exposed on $e\left(v_{i},v_{j}\right)$ during time period p. Therefore, the risk on $e\left(v_{i},v_{j}\right)$ is $r_{ij}^{p}=P_{ij}^{p}\lambda pop_{ij}^{p}a_{ij}$. The accident rate $P_{ij}^{p}$ can be obtained from historical statistical data. The population data $pop_{ij}^{p}$ for different time periods can be accessed through platforms such as the Baidu Maps Huiyan City Population Geographic Data Platform, among others.

### Vehicle travel cost and overtime penalty cost calculation

The total cost of the delivery process consists of two components: Transportation cost incurred during vehicle movement, penalty cost incurred when the delivery exceeds the required time. The total transportation cost for the first component can be expressed as $min\sum_{i=0}^{n}\sum_{j=0}^{n}\sum_{k=1}^{k}c\mu_{ij}x_{ijk}$. For the second component, Fig 1 illustrates the penalty cost function, where the horizontal axis represents the arrival time, and the vertical axis represents the penalty cost. If the vehicle arrives before or on time, i.e., $A_{ik}\leq W_{i}$, no penalty is incurred. However, if the vehicle arrives later than required, i.e., $A_{ik}>W_{i}$, it is considered a delayed service, and the penalty cost increases with the delay time. Thus, the total penalty cost can be expressed as $\sum_{i=1}^{n}c^{\prime }max\left\{\left(\sum_{k=1}^{k}A_{ik}-W_{i}\right),0\right\}$.

**Fig 1:**
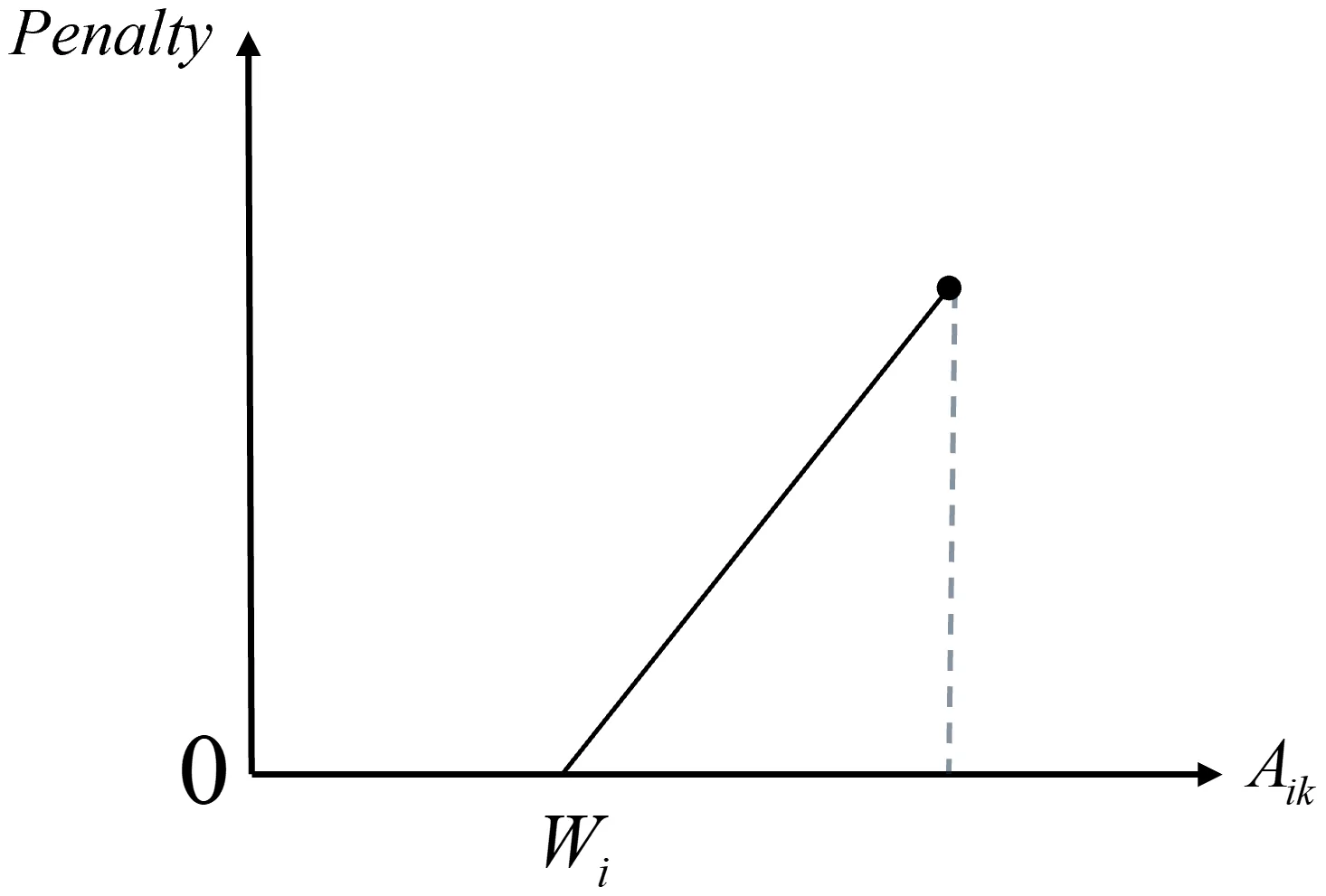
The illustration of penalty cost.

### Model construction


$$\begin{array}{c} minf_{\text{1}}=min\sum_{i=0}^{n}\sum_{j=0}^{n}\sum_{k=1}^{K}c\mu_{ij}x_{ijk}+\sum_{i=1}^{n}c^{\prime }max\left\{\left(\sum_{k=1}^{k}A_{ik}-W_{i}\right),0\right\} \end{array}$$ (1)

$$\begin{array}{c} minf_{\text{2}}=min\sum_{p=1}^{P}\sum_{i=0}^{n}\sum_{j=1}^{n}\sum_{k=1}^{k}P_{ij}^{p}\lambda pop_{ij}^{p}a_{ij}z_{ik}^{p} \end{array}$$ (2)

s.t


$$\begin{array}{c} \sum_{j=0}^{n}x_{jik}=\sum_{j=0}^{n}x_{ijk}\text{(}i=0,1,...,n;k=1,2,...,K) \end{array}$$ (3)

$$\begin{array}{c} \sum_{j=0}^{n}x_{ijk}=y_{ik}\text{(}i=1,2,...,n;k=1,2,...,K) \end{array}$$ (4)

$$\begin{array}{c} \sum_{k=1}^{K}y_{0k}=K \end{array}$$ (5)

$$\begin{array}{c} \sum_{k=1}^{K}y_{ik}=1\text{(}i=1,2,...,n;k=1,2,...,K) \end{array}$$ (6)

$$\begin{array}{c} u_{0k}=0\;(k=1,2,...,K) \end{array}$$ (7)

$$\begin{array}{c} u_{jk}+q_{i}-u_{ik}\leq (1-x_{ijk})M\;(i=1,2,...,n;j=0,1,...,n;k=1,2,...,K) \end{array}$$ (8)

$$\begin{array}{c} u_{jk}-a_{ij}\leq (1-x_{ijk})M\;(i=0,1,...,n;j=1,2,...,n;k=1,2,...,K) \end{array}$$ (9)

$$\begin{array}{c} d_{0k}\geq w_{0k}\;(k=1,2,...,K) \end{array}$$ (10)

$$\begin{array}{c} d_{ik}\geq A_{ik}+w_{ik}\;(i=1,2,...,n;k=1,2,...,K) \end{array}$$ (11)

$$\begin{array}{c} d_{ik}+t_{ij}-A_{jk}\leq (1-x_{ijk})M\;(i=0,1,...,n;j=1,2,..,n;k=1,2,...,K) \end{array}$$ (12)

$$\begin{array}{c} \sum_{p=1}^{D}z_{ik}^{p}=\sum_{j=1}^{n}x_{ijk}\;(i=0,1,...,n;k=1,2,...,K) \end{array}$$ (13)

$$\begin{array}{c} a^{p}z_{ik}^{p}\leq d_{ik}<b^{p}z_{ik}^{p}+(1-z_{ik}^{p})M\;(i=0,1,...,n;k=1,2,...,K;p=1,2,...,D) \end{array}$$ (14)

$$\begin{array}{c} u_{ik}\leq Q\;(i=1,2,...,n;k=1,2,...,K) \end{array}$$ (15)

$$\begin{array}{c} x_{ijk},y_{ik},z_{ik}^{p}\in \left\{0,1\right\},a_{ij},u_{ik},A_{ik},d_{ik},w_{ik}\geq 0\;(i=0,1,...,n;j=0,1,...,n;k=1,2,...,K;p=1,2,...,D) \end{array}$$ (16)

The objective function (1) minimizes the total transportation cost. The objective function (2) minimizes the total transportation risk, specifies only when vehicle k departs from node $v_{i}$ and enters edge $\left(v_{i},v_{j}\right)$ during time period p (i.e., $z_{ik}^{p}=1$), does this edge account for the population exposure $pop_{ij}^{p}$ of that time period. For other time periods $z_{ik}^{p}=0$, this term evaluates to zero and is not included in the objective function. In objective function (2), $z_{ik}^{p}$ is a binary variable that equals 1 if vehicle k departs from node $v_{i}$ during time period p, and 0 otherwise. Since the vehicle enters edge $\left(v_{i},v_{j}\right)$ immediately after departing from node $v_{i}$, $z_{ik}^{p}$ simultaneously determines the time period to which the traversal of edge $\left(v_{i},v_{j}\right)$ belongs. For any edge $\left(v_{i},v_{j}\right)$, its risk value $P_{ij}^{p}\lambda pop_{ij}^{p}a_{ij}$ is included in the total risk if and only if the edge is actually traversed and the traversal occurs in time period p. If the edge is not traversed by vehicle k, constraint (13) ensures that $z_{ik}^{p}=0$ for all p; if the vehicle traverses the edge in time period p, only the corresponding p -term is activated, while all other period terms vanish. Constraint (3) ensures that each vehicle must leave each demand point after visiting it and return to the distribution center at the end of the route. Constraint (4) stipulates that a vehicle can only visit points that lie along its planned route. Constraint (5) defines the number of vehicles available at the distribution center. Constraint (6) ensures that each customer is visited only once. Constraints (7)-(9) describe the variations in vehicle load. Constraints (10)-(11) define the relationship between departure times, arrival times, and waiting times at nodes. Constraint (12) specifies that if vehicle k travels directly from node $v_{i}$ to node $v_{j}$, then the arrival time $A_{jk}$ of vehicle k at node $v_{j}$ must be at least the departure time $d_{ik}$ from node $v_{i}$ plus the travel time $t_{ij}$ on edge $\left(v_{i},v_{j}\right)$. If the vehicle does not traverse edge $\left(v_{i},v_{j}\right)$, this constraint is relaxed and becomes non-binding. When $x_{ijk}=1$, constraint (12) yields $A_{jk}\geq d_{ik}+t_{ij}$, together with constraint (11), $A_{jk}\geq A_{ik}+t_{ij}>A_{ik}$, since $t_{ij}>0$ for all edges, the arrival times along any vehicle route are strictly increasing. If there existed a closed subtour not passing through the depot $v_{\text{0}}$, say $v_{\text{1}}\to v_{\text{2}}\to ...\to v_{m}\to v_{\text{1}}$, then applying the above inequality successively along this subtour would yield $A_{\text{1}}<A_{\text{2}}<...A_{m}<A_{\text{1}}$, a contradiction. Hence, no closed subtour can exist. Constraint (13) specifies that if vehicle k departs from node $v_{i}$, it must choose exactly one departure time period; if vehicle k does not depart from node $v_{i}$, no time period is selected. Constraint (14) indicates that when $z_{ik}^{p}=1$, i.e., when period p is selected, the departure time $d_{ik}$ must lie within the range $\left[a^{p},b^{p}\right)$. Constraint (15) is the vehicle load capacity constraint, and constraint (16) defines the range of the variables.

## Algorithm implementation

The optimization of transportation routes for hazardous materials in urban environments, based on time-varying road networks, constitutes a complex multi-objective problem that is classified as NP-hard. Given the inherent complexity, exact algorithms are often impractical for large-scale instances due to the prohibitive computational time required to obtain solutions. In this section, we initially introduce an exact algorithm utilizing the Gurobi solver to solve an instance involving 10 nodes. Subsequently, we elaborate on the design of an advanced approximation algorithm, namely PPO-MOEAD, specifically tailored for solving instances of progressively increasing sizes, encompassing 10, 50, 75, and 100 nodes, thus offering a pragmatic alternative for larger, more computationally demanding scenarios. Table 5 shows the parameters of this section.

**Table 5:** Parameters of algorithm designing section.

Symbol	Definition
(v,p)	A node-time period decision
$\tau_{risk}$	Risk growth rate
r(p)	Risk value at node during time period p
$\omega_{\text{1}}$	Risk growth threshold, used to determine whether a decision can be constantized
$\varpi^{*}$	Optimal rule parameter
I(v,p)	Importance of decision (v,p), defined as the loss in the objective value when the waiting time of this decision is fixed to zero
ε	Importance threshold; decisions with importance below this threshold are identified as non-critical
Num(ε)	Number of decisions that are constantized
ϖ	Rule parameter vector, consisting of learnable parameters such as the risk growth threshold
$Z^{*}$	Optimal solution to the original problem
$Z^{\prime }$	Simplified solution obtained by fixing the waiting times of non-critical decisions to zero
$Z^{*}|x_{v,p=w_{ik}}$	New solution obtained by replacing the value of decision (v,p) in $Z^{*}$ with $w_{ik}$
ΔF	Loss in the objective function value due to simplification
L(v,p)	Estimated loss of benefit for decision (v,p)
$bene_{o}$	Actual net benefit of a decision point in the high-quality solution
$bene_{a}$	Net benefit recalculated after forcibly setting the waiting time of the decision point to zero
Tni	Total number of iterations for which the MOEA/D algorithm is run
$HQ_{s}$	High-quality Pareto front solution set collected by running MOEA/D
$S_{fixed}\left(\varpi \right)$	Set of decisions that are constantized under the rule parameter ϖ
$\hat{\Delta }F\left(\varpi \right)$	Estimated total loss under the rule parameter ϖ
${ℜ}_{\text{1}}$	Search interval for the risk growth threshold$\omega_{\text{1}}$
$R\left(\varpi^{*}\right)$	Optimal rule for determining which decisions should be constantized
ζ	Bounded bias term arising from fitting errors in historical data
ψ	Total number of decision variables in the original problem (waiting time decisions at all nodes)
J	Constantization marking table, indicating whether each decision is constantized
$\upsilon_{f}$	Set of constantized variables (waiting times fixed to zero)
$\upsilon_{o}$	Set of variables to be optimized (not constantized)
$S_{t}$	State space
$U_{t}$	Set of unsatisfied customer nodes
$cur_{k}$	Current vehicle number
$pos_{c}$	Current position
$cur_{l}$	Current vehicle load
$cur_{t}$	Current time
$cur_{p}$	Current time period
$can_{i}$	Candidate customer nodes
$dis_{i}$	Distance to the candidate customer
$Act_{t}$	Action state
$arr_{i}$	Arrival time at the candidate customer
$acts_{c}$	Action selecting the next customer to serve
$actd_{w}$	Waiting decision action
$actc_{d}$	Route control action
$Rew_{t}$	Reward function
$ri_{p}$	Path risk penalty
$dis_{p}$	Distance cost penalty
$tw_{p}$	Time window penalty
$wd_{r}$	Waiting decision reward
$cv_{p}$	Constraint violation penalty
θ	PPO policy network parameters
ϕ	PPO value network parameters
γ	Discount factor
η	Learning rate
$\varepsilon_{clip}$	Clipping factor
$ad_{t}$	Advantage function
N	Number of subproblems
$\lambda^{h}$	Weight vector
Wv	Set of all weight vectors
$x^{h}$	Solution corresponding
IP	Initial population
T	Neighborhood size
B(h)	Neighborhood set
$g^{h}\left(x\right)$	Scalarized objective function
m	Number of objectives
$\lambda_{i}^{h}$	Weight coefficient
$evo_{p}$	Evolutionary progress
$div_{p}$	Population diversity indicator
$sta_{f}$	Stagnation flag
$sim_{p}$	Similarity between two parent solutions
$diff_{p}$	Quality difference between two parent solutions
$cur_{ou}$	Frequency of current operator usage
$cro_{o}$	Crossover operator selection
$mut_{o}$	Mutation operator selection
$act_{t}$	Action space
$rew_{q}$	Quality improvement reward
$rew_{d}$	Diversity reward
$rew_{p}$	Stagnation breakthrough reward
$rew_{s}$	Stage adaptability reward
$z_{m}$	Ideal value in the current population
$iv_{cp}$	Crossover probability
$mp_{iv}$	Mutation probability
$amp_{m}$	Mutation amplitude
$\pi_{\theta }\left(S_{t}\right)$	Action probability distribution output by the policy network

### Exact algorithm

This section presents an exact solution approach based on Mixed-Integer Linear Programming (MILP). Directly solving the complete MILP model becomes computationally expensive and time-consuming when the number of variables is large. To enhance both the feasibility and efficiency of the exact solution, we propose a model preprocessing technique, Node Departure Time Decision Recognition Optimization. This strategy involves the identification of specific departure time decision variables and their constant assignment prior to the commencement of the solution process, thereby effectively reducing the search space and improving computational efficiency.

#### Node departure time decision recognition optimization.

In the vehicle routing problem considering time-varying risks, the computation time and complexity increase significantly as the number of customer nodes and time periods grows. Traditional solution methods require optimization of all these continuous variables, leading to high computational complexity and inefficiency. Through the analysis of training data, it has been observed that in real-world delivery processes, there exists a considerable number of “never waiting” or extremely low-waiting-reward” points. The optimization of these non-critical decisions results in a waste of computational resources. By identifying and fixing these non-essential departure time decisions to constant values, the computational complexity can be significantly reduced, leading to more efficient solutions.

The framework for identifying non-critical departure time decisions is based on the following rule, which evaluates the context of a “node-time period” decision (v,p) and outputs a binary determination: either constantization (setting the departure time decision to a fixed value), forcing the waiting time to be zero, or retaining the original decision. The key threshold for decision-making within this rule is treated as a trainable parameter, thus transforming the learning problem of the constantization strategy into a low-dimensional parameter optimization problem.

If, upon arriving at a customer node, the increase in risk for the upcoming time period is minimal, the decision is made to serve the customer immediately in the current time period, thus avoiding waiting. The additional risk exposure incurred by this decision is deemed acceptable, and the departure time decision can be constantized. The risk growth rate $\tau_{risk}$ can be defined as $\tau_{risk}=\frac{r(p+1)-r\left(p\right)}{r\left(p\right)}$, where r(p) represents the risk value at node during time period p. If $\tau_{risk}<\omega_{\text{1}}$, the decision is constantized, where $\omega_{\text{1}}$ is the risk growth rate threshold to be learned.

For any given decision (v,p), as long as the risk remains stable, that decision is marked as constantizable. The entire constantization strategy is determined by the parameter vector $\varpi =\left(\omega_{\text{1}}\right)$. The problem of learning the optimal constantization strategy is then transformed into finding the parameter combination $\varpi^{*}$ that optimizes the overall objective.

To provide a theoretical objective for the learning of rule parameters, we first formalize the “importance” of decisions. Proposition 4−1 is presented as follows: Proposition 4−1 states that constantizing all decisions satisfying I(v,p)<ε results in an upper bound on the total loss of mass of ∑I(v,p)≤Num(ε)·ε. In other words, the objective is to learn the rule parameters $\varpi^{*}$ such that the sum of the importance of the decisions in the set that are constantized by the rule is minimized as much as possible.

**Proposition 4−1: Upper Bound on the Quality of Decision Constantization:** Let Z be an optimal solution to the hazardous materials vehicle routing problem based on time-varying road networks, with the corresponding objective value F(Z). For any waiting decision at node $v_{i}$ and time period p, denoted as (v,p), its importance is defined as $I(v,p)=\min_{w_{ik}\in [0,w_{\max}]}\left[F(Z^{*})-F(Z^{*}|x_{v,p=w_{ik}})\right]$. In this context, $Z^{*}|x_{v,p=w_{ik}}$ represents the solution obtained by replacing the value of the decision (v,p) in $Z^{*}$ with $w_{ik}$, resulting in a new decision configuration. If we construct a simplified solution $Z^{\prime }$, we define: If I(v,p)<ε, then set ${Z^{\prime }}_{v,p}=0$; If I(v,p)≥ε, then retain the original value ${Z^{\prime }}_{v,p}={Z^{*}}_{v,p}$. In other words, for decisions with an importance below the threshold ε, we set their waiting time to zero (i.e., no waiting at the customer node), while retaining the original values for all other decisions. The increase in the objective function value due to simplification is denoted as $\Delta F=F\left(Z^{\prime }\right)-F\left(Z^{*}\right)\leq \sum_{\left(v,p\right):I\left(v,p\right)<\varepsilon }I\left(v,p\right)\leq Num\left(\varepsilon \right)\cdot \varepsilon$, where Num(ε)=|{(v,p)|I(v,p)<ε| represents the number of decisions that have been simplified.

We employ a two-stage optimization process to learn the optimal parameters $\varpi^{*}$. First, the standard MOEA/D algorithm is run for Tni iterations, collecting a high-quality Pareto front solution set $HQ_{s}$. Each solution in $HQ_{s}$ includes the complete path, arrival times at each node, and the actual waiting times at each node. For each point, we calculate its loss of benefit L(v,p) as an approximate estimate of I(v,p), defined as: $L\left(v,p\right)=max(0,bene_{o}-bene_{a})$, where $bene_{o}$ is the actual net benefit of that point in the high-quality solution, and $bene_{a}$ is the net benefit recalculated by forcibly setting the waiting time of that point to zero and reassessing through local simulation.

Next, we define the estimated total loss under the parameterized rule. For any parameter ϖ, when applied to the historical dataset, a set of constantized decisions $S_{fixed}\left(\varpi \right)$ is obtained. The estimated total loss under this rule parameter is defined as $\hat{\Delta }F\left(\varpi \right)=\sum_{\left(v,p\right)\in S_{fixed}\left(\varpi \right)}L\left(v,p\right)$, where $\hat{\Delta }F\left(\varpi \right)$ reflects the estimated upper bound of theoretical quality loss after applying the rule. Finally, parameter optimization is performed using grid search. The learning objective is transformed into a two-dimensional single-objective optimization problem, i.e., $\varpi^{*}=arg\;\underset{\varpi }{min\hat{\Delta }F\left(\varpi \right)}$. For each parameter, a range and step size are set. The risk growth threshold $\omega_{\text{1}}$ is within the interval ${ℜ}_{\text{1}}$. All combinations are traversed, and the optimal $\varpi^{*}$ that minimizes $\hat{\Delta }F\left(\varpi \right)$ is selected. Based on this learning process, we can derive Proposition 4−2.

**Proposition 4−2: Optimality of the Rule Parameters:** Let $\varpi^{*}$ be the optimal rule parameter obtained through the above process, corresponding to the minimal estimated loss $\hat{\Delta }F\left(\varpi^{*}\right)$. For new instances with the same decision pattern distribution as the historical data, after applying the rule $R\left(\varpi^{*}\right)$ for decision constantization, the resulting expected quality loss is denoted as $E[\Delta F]\leq \hat{\Delta }F\left(\varpi^{*}\right)+\zeta$, where ζ represents a bounded bias term arising from fitting errors in the historical data. This proposition indicates that the process of minimizing $\hat{\Delta }F\left(\varpi \right)$ directly optimizes the worst-case performance upper bound of the constantized decisions on new problem instances.

After initializing the model in the solver and before starting the optimization process, we perform the decision constantization strategy for preprocessing. For a given instance, we iterate over all ψ possible decisions (v,p), applying $R\left(\varpi^{*}\right)$ to make a judgment and generating a constantization marking table J. Based on this table, all decision variables are divided into two mutually exclusive sets: one is the constantized variable set $\upsilon_{f}$, which contains variables that satisfy any rule in $R\left(\varpi^{*}\right)$, and for these variables, their waiting time is permanently fixed to 0 for the entire subsequent optimization process. The other set is the set of variables to be optimized, $\upsilon_{o}$, which contains variables that do not satisfy any constantization rule and thus represent the decision space for the subsequent algorithm. This preprocessing is performed only once, and its output is a problem where the dimensionality is reduced from ψ to $\left|\upsilon_{o}\right|$. Subsequently, throughout the entire algorithm, all decision variables marked as constantized will have their values fixed to 0.

#### Comparison of solution time.

Fig 2 illustrates the solution results for 8 typical instances. After applying the Node Departure Time Decision Recognition Optimization strategy, the number of waiting times in the model is reduced, leading to an average reduction of 26% in the solution time.

**Fig 2:**
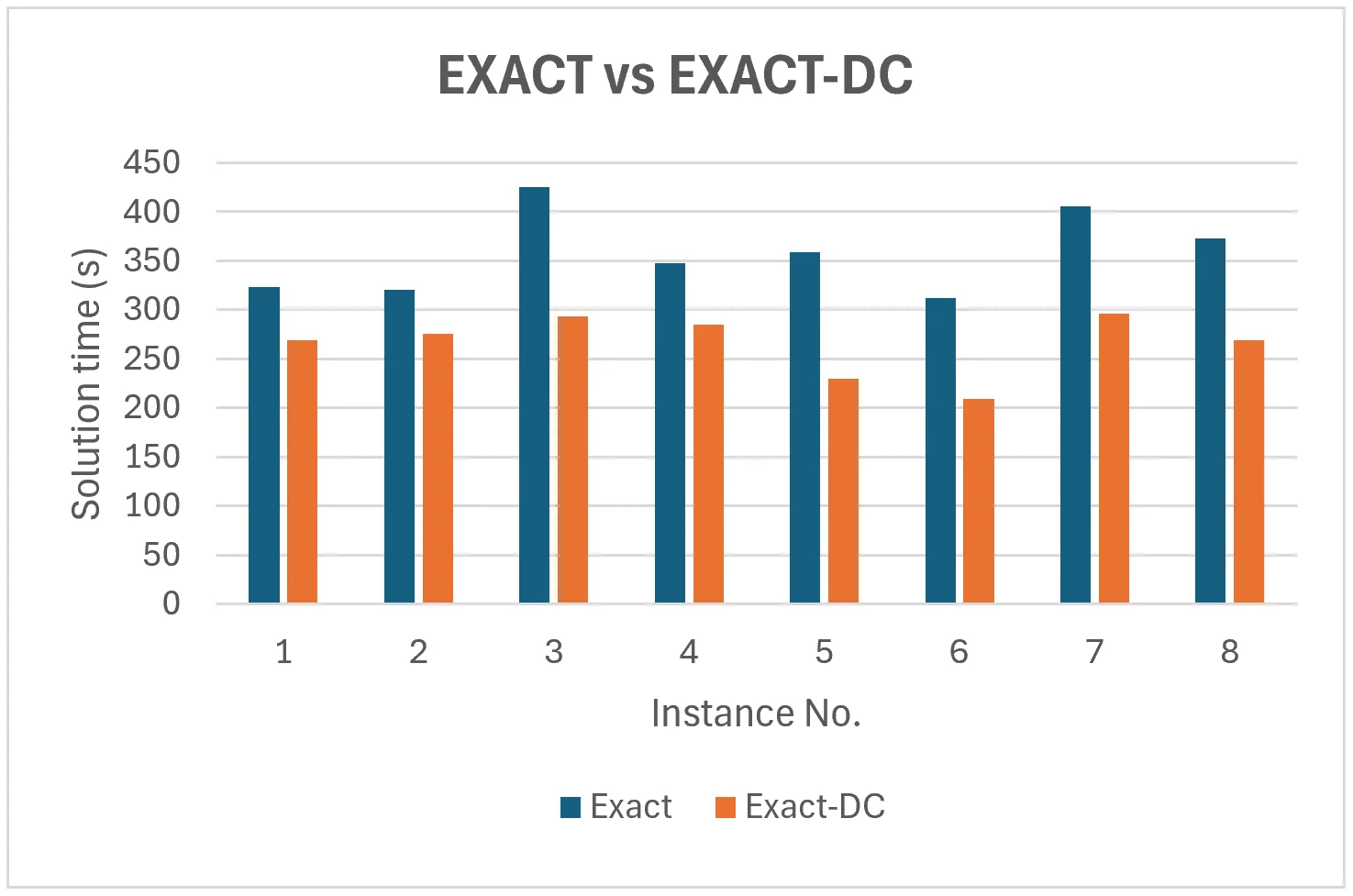
Comparison of solution time between exact algorithm and improved exact algorithm.

### Reinforcement learning-based decomposed multi-objective evolutionary algorithm (PPO-MOEA/D)

To address medium- to large-scale multi-objective transportation route optimization problems, the Decomposed Multi-Objective Evolutionary Algorithm (MOEA/D), originally proposed by Zhang & Li, has demonstrated exceptional performance [43]. This method decomposes the aggregation function of a multi-objective optimization problem into a series of single-objective optimization subproblems, optimizing each subproblem via evolutionary operations in conjunction with its neighboring subproblems. Over time, significant enhancements have been made to this approach, where the optimal solution for each subproblem corresponds to a Pareto optimal solution for the overall multi-objective optimization problem. To better align the solution with the unique characteristics of this specific problem, we integrate a Departure Time Decision Recognition Optimization as a preprocessing step for the instances and leverage the PPO (Proximal Policy Optimization) reinforcement learning method to augment the decomposed multi-objective evolutionary algorithm. The following outlines three key improvements:

(1) A preprocessing rule based on risk and time features, namely Node Departure Time Decision Recognition Optimization, has been designed. By leveraging historical data, the optimal threshold for the rule is learned, enabling the identification and constantization of a set of non-critical departure time decision variables, thereby reducing the optimization search space.
(2) The PPO policy network is used to generate a high-quality initial population based on the problem’s characteristics, replacing the traditional MOEA/D approach of randomly or uniformly generating initial solutions. This provides a good starting point for the evolutionary algorithm with both diversity and convergence, accelerating the early-stage convergence process.
(3) The PPO policy network is utilized to dynamically select the optimal combination of crossover and mutation operators based on the current population state. This adaptive adjustment of the search strategy enhances the algorithm’s adaptability across different search stages, improving overall performance.

For ease of explanation, we use an example that includes a distribution center $v_{\text{0}}$ and six customers $v_{\text{1}},\cdots ,v_{\text{6}}$. The coordinates of the network’s nodes are randomly generated within the range [0, 50], and the demand for each customer is randomly generated within the range [10, 15]. It is assumed that the vehicle speed on each edge is 1. The example consists of 3 vehicles and 3 time periods, each with a duration of 20. The accident rate on each edge for each time period is randomly generated within the range [1%, 6%], and the population on each edge is randomly generated within the range [1, 100]. The coordinates and demand of the customers are shown in Table 6, and the travel time matrix for each edge is shown in Table 7.

**Table 6:** Customer coordinates and demand.

Customer ID	X Coordinate	Y Coordinate	Demand
*v*_0_	0	0	–
*v*_1_	10	20	15
*v*_2_	15	25	13
*v*_3_	18	21	10
*v*_4_	25	20	11
*v*_5_	30	15	14
*v*_6_	35	10	15

**Table 7:** Travel time matrix.

Customer ID	*v*_0_	*v*_1_	*v*_2_	*v*_3_	*v*_4_	*v*_5_	*v*_6_
*v*_0_	0	22.36	29.15	27.66	32.02	33.54	36.40
*v*_1_	22.36	0	7.07	8.06	15	22.36	26.93
*v*_2_	29.15	7.07	0	5	10.30	18.03	23.54
*v*_3_	27.66	8.06	5	0	7.07	14.32	19.72
*v*_4_	32.02	15	10.30	7.07	0	7.07	11.18
*v*_5_	33.54	22.36	18.03	14.32	7.07	0	7.07
*v*_6_	36.40	26.93	23.54	19.72	11.18	7.07	0

#### Encoding and decoding.

To accommodate the solution requirements of this problem, a hybrid encoding scheme is designed. The encoding consists of 15 bits: [2,6,5,1,3,4,0,0,16,0,0,0,0,8,0], the first 6 bits represent the customer numbers [2,6,5,1,3,4], corresponding to the sequential visits to customers 2–4. The next 3 bits represent the departure times of the vehicles from the distribution center $v_{\text{0}}$, where the vehicle numbers 1–3 have respective waiting times of 0, 0, and 16 minutes, denoted as [0,0,16]. The last 6 bits represent the waiting times required for the vehicles at customers 1–6, denoted as [0,0,0,0,8,0]. To simplify the encoding structure and improve algorithm efficiency, a pre-processing step based on the departure time decision recognition optimization is applied to the original encoding. Specifically, the waiting time decisions for customers 2 and 6 are permanently removed, as they are identified as non-critical. This reduces the encoding length from 15 bits to 13 bits, while retaining only the waiting times at critical customer nodes. A 13-bit fixed-length encoding is used to represent the complete solution to the vehicle routing problem. The first 6 bits correspond to the customer numbers [2,6,5,1,3,4], and the next 3 bits represent the departure times of the vehicles from the distribution center $v_{\text{0}}$, given as [0,0,16]. The first two segments of the encoding remain unchanged. The last 4 bits represent the allowed waiting times at the critical customer nodes, indicating the waiting times at customers 1, 3, 4, and 5, which are [0,0,0,8] minutes, respectively. The encoding and decoding process is illustrated in Fig 3.

**Fig 3:**
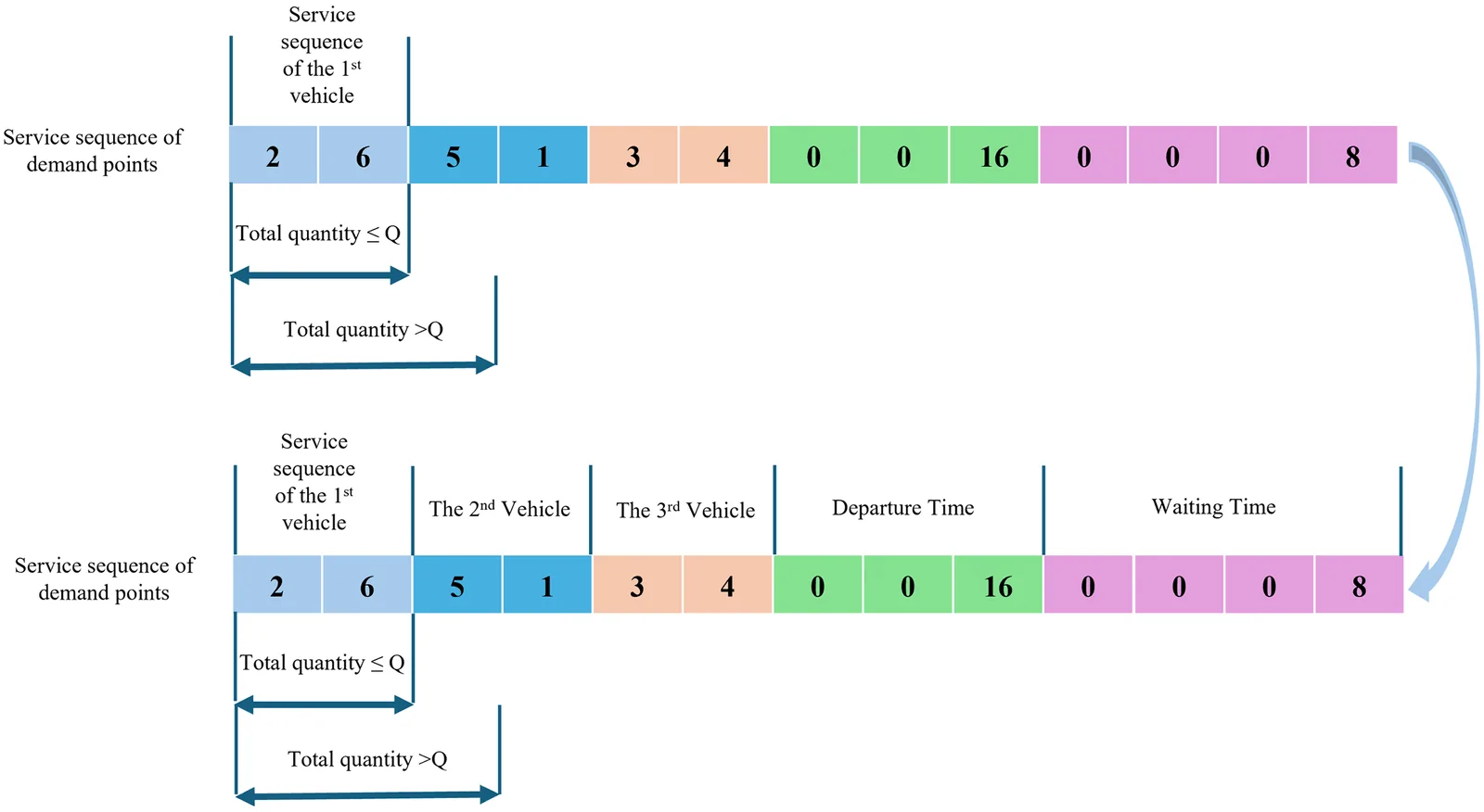
Encoding and decoding illustration.

The illustrations of vehicle route risks, considering node waiting and not considering node waiting, are shown in Figs 4 and 5, respectively.

**Fig 4:**
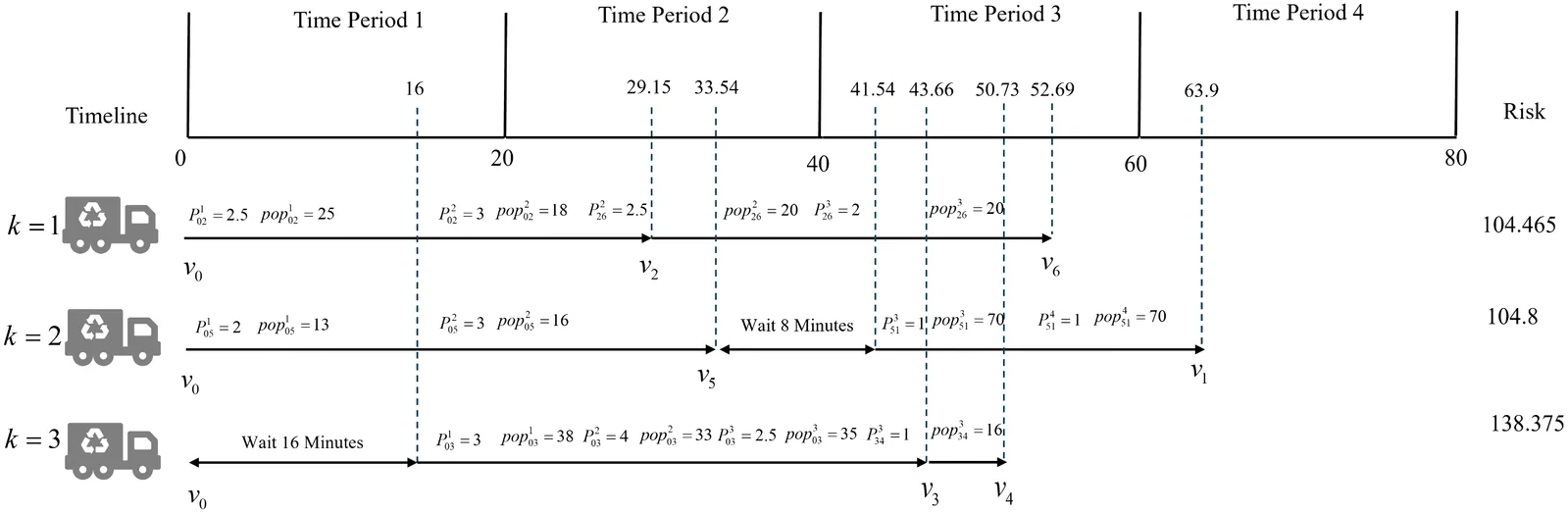
Illustration of vehicle route risk considering node waiting.

**Fig 5:**
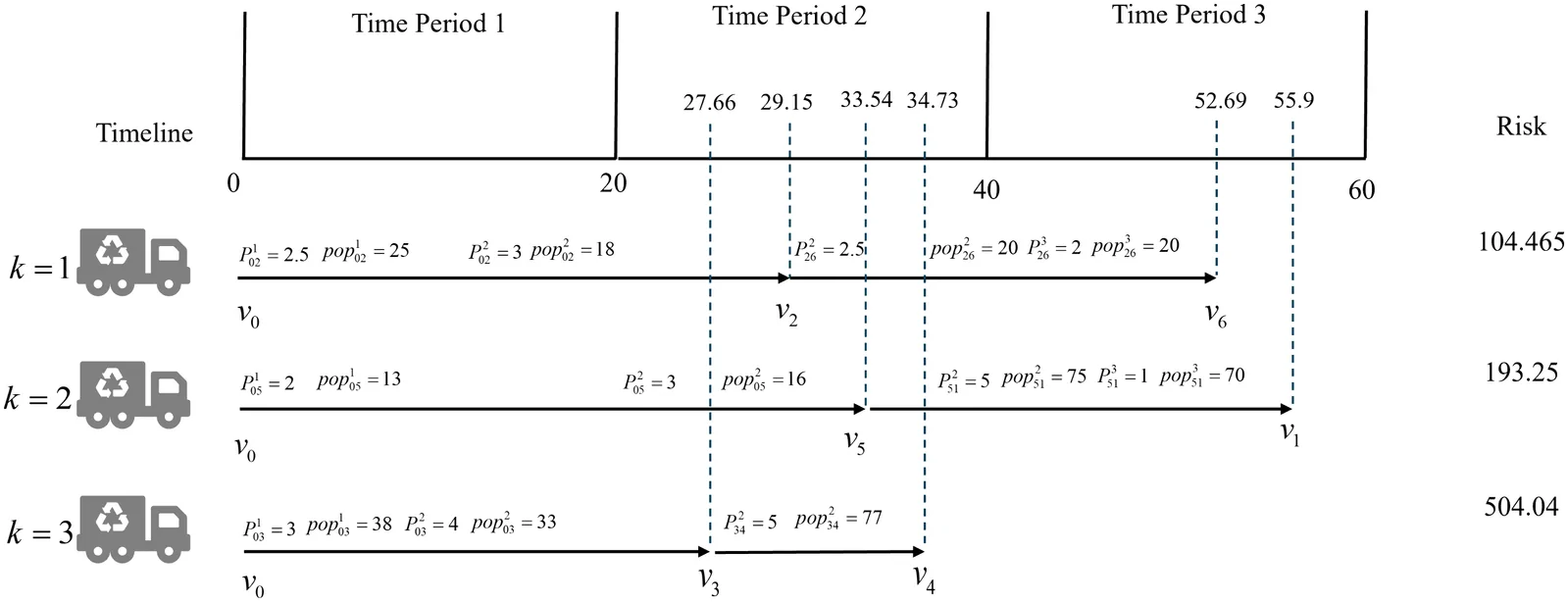
Illustration of vehicle route risk without considering node waiting.

The path for vehicle k=1 is $v_{\text{0}}\to v_{\text{2}}\to v_{\text{6}}\to v_{\text{0}}$, with no waiting required at any of the nodes. The risk calculation is as follows: $P_{02}^{\text{1}}\times pop_{02}^{\text{1}}\times 0.69=2.5\times 25\times 0.69=43.125$; $P_{02}^{\text{2}}\times pop_{02}^{\text{2}}\times \text{(}1-0.69\text{)}=3\times 18\times \text{(}1-0.69\text{)}=16.74$; $P_{26}^{\text{2}}\times pop_{26}^{\text{2}}\times 0.46=2.5\times 20\times 0.46=23$; $P_{26}^{\text{3}}\times pop_{26}^{\text{3}}\times \text{(}1-0.46\text{)}=2\times 20\times \text{(}1-0.46\text{)}=21.6$, resulting in a risk of 43.125+16.74++23+21.6=104.465 and a cost of 29.15+23.54+36.4=89.09.

The path for vehicle k=2 is $v_{\text{0}}\to v_{\text{5}}\to v_{\text{1}}\to v_{\text{0}}$, where it waits for 8 minutes at customer 5, transitioning into time period 3. The risk calculation is as follows: $P_{05}^{\text{1}}\times pop_{05}^{\text{1}}\times 0.6=2\times 13\times 0.6=15.6$; $P_{05}^{\text{2}}\times pop_{05}^{\text{2}}\times (1-0.6)=3\times 16\times (1-0.6)=19.2$; $P_{51}^{\text{3}}\times pop_{51}^{\text{3}}\times 0.83=1\times 70\times 0.83=58.1$; $P_{51}^{\text{4}}\times pop_{51}^{\text{4}}\times (1-0.83)=1\times 70\times (1-0.83)=11.9$, resulting in a risk of 15.6+19.2+58.1+11.9=104.8 and a cost of 33.54+22.36+22.36=78.26. If no waiting occurs, the risk calculation is as follows: $P_{05}^{\text{1}}\times pop_{05}^{\text{1}}\times 0.6=2\times 13\times 0.6=15.6$; $P_{05}^{\text{2}}\times pop_{05}^{\text{2}}\times (1-0.6)=3\times 16\times (1-0.6)=19.2$; $P_{51}^{\text{2}}\times pop_{51}^{\text{2}}\times 0.29=5\times 75\times 0.29=108.75$; $P_{51}^{\text{3}}\times pop_{51}^{\text{3}}\times 0.71=1\times 70\times 0.71=49.7$, resulting in a risk of 15.6+19.2+108.75+49.7=193.25 and a cost of 33.54+22.36+22.36=78.26.

The path for vehicle k=3 is $v_{\text{0}}\to v_{\text{3}}\to v_{\text{4}}\to v_{\text{0}}$, where it waits for 16 minutes at node 0, transitioning into time period 3. The risk calculation is as follows: $P_{03}^{\text{1}}\times pop_{03}^{\text{1}}\times 0.14=3\times 38\times 0.14=15.96$; $P_{03}^{\text{2}}\times pop_{03}^{\text{2}}\times 0.72=4\times 33\times 0.72=95.04$; $P_{03}^{\text{3}}\times pop_{03}^{\text{3}}\times 0.13=2.5\times 35\times 0.13=11.375$; $P_{34}^{\text{3}}\times pop_{34}^{\text{3}}=1\times 16=16$, resulting in a risk of 15.96+95.04+11.375+16=188.375 and a cost of 27.66+7.07+32.02=66.75. If no waiting occurs, the risk calculation is as follows: $P_{03}^{\text{1}}\times pop_{03}^{\text{1}}\times 0.72=3\times 38\times 0.72=82.08$; $P_{03}^{\text{2}}\times pop_{03}^{\text{2}}\times 0.28=4\times 33\times 0.28=36.96$; $P_{34}^{\text{2}}\times pop_{34}^{\text{2}}=5\times 77=385$, resulting in a risk of 82.08+36.96+385=504.04 and a cost of 27.66+7.07+32.02=66.75.

In summary, the total cost is represented by objective function 1, $f_{\text{1}}=89.09+78.26+66.75=234.1$, and the total risk is represented by objective function 2, $f_{\text{2}}=104.465+104.8+138.375=347.64$.

(1) **PPO-based initial solution generation and evaluation**
  1) **PPO-improved initial solution strategy design**

The generation of the initial solution needs to be based on the characteristics of the departure times, while considering constraints such as time windows for delivery routes, traffic conditions, customer demands, and real-time risk factors. To enhance the quality of the initial solution, a PPO algorithm is used to improve the random generation of initial solutions in the MOEA/D algorithm. This approach determines the initial loading of paths and checks whether customer nodes in the randomly generated paths require adjustments. The goal is to improve the overall quality of the initial solution. Below are the state space, action space, and reward function for PPO applied to the initial solution.

The state space includes the set of unsatisfied customer nodes $U_{t}$, the current vehicle number $cur_{k}$, the current position $pos_{c}$, the current load $cur_{l}$, the current time $cur_{t}$, the current time period $cur_{p}$, the candidate customer nodes $can_{i}$, the distance to the candidate customer $dis_{i}$, and the arrival time at the candidate customer $arr_{i}$, denoted as $S_{t}=\left\{U_{t},cur_{k},pos_{c},cur_{l},cur_{t},cur_{p},can_{i},dis_{i},arr_{i}\right\}$. The action space includes selecting a customer as the next service node $acts_{c}$, waiting decision action $actd_{w}$, and route control action $actc_{d}$, denoted as $Act_{t}=\left\{acts_{c},actd_{w},actc_{d}\right\}$. The reward function includes the path risk penalty $ri_{p}$, the distance cost penalty $dis_{p}$, the time window penalty $tw_{p}$, the waiting decision reward $wd_{r}$, and the constraint violation penalty $cv_{p}$, denoted as $Rew_{t}=\left\{Ri_{p},dis_{p},Tw_{p},Wd_{r},Cv_{p}\right\}$.

**Table:** 

Algorithm 1: PPO Algorithm-Enhanced MOEA/D for Initial Solution Generation
Step 1. Initialization. Generate random customer demands and vehicle capacity constraints. If the next customer exceeds the current vehicle’s capacity, add a new vehicle. Generate the initial vehicle set, routing set, dispatch load, and risk intervals. Initialize the PPO network with policy parameters θ, value network parameters ϕ, discount factor γ, learning rate η, and clipping factor $\varepsilon_{clip}$.
Step 2. Action Sampling and Execution. Define the current state $S_{t}$. Use the policy network to sample an action $act_{t}$. Reallocate customer points and update the routes.
Step 3. Reward Calculation. Compute the total reward function $Total_{R}$.
Step 4. Policy Update. Record the state $S_{t}$, action $Act_{t}$, reward $Rew_{t}$, and next state $S_{t+1}$. Update the policy network using PPO loss optimization and the advantage function $ad_{t}$.
Step 5. Evolutionary Optimization. Repeat steps 2–4 until the stopping condition is met. Optimize route loading and customer allocations by minimizing the reward function.

2) **PPO-improved random initial solution**

After optimizing the random initial solution using the PPO strategy, the path for vehicle k=1 is $v_{\text{0}}\to v_{\text{1}}\to v_{\text{2}}\to v_{\text{0}}$, where it waits for 18 minutes at customer 1, transitioning into time period 3. The risk calculation is as follows: $P_{01}^{\text{1}}\times pop_{01}^{\text{1}}\times 0.894=1\times 21\times 0.894=18.774$; $P_{01}^{\text{2}}\times pop_{01}^{\text{2}}\times \text{(}1-0.894\text{)}=2\times 13\times \text{(}1-0.894\text{)}=2.756$; $P_{12}^{\text{3}}\times pop_{12}^{\text{3}}=1\times 65=65$, resulting in a risk of 18.774+2.756+65=86.53 and a cost of 22.36+7.07+29.15=58.58.

The path for vehicle k=2 is $v_{\text{0}}\to v_{\text{5}}\to v_{\text{3}}\to v_{\text{0}}$, with no waiting throughout the route. The risk calculation is as follows: $P_{05}^{\text{1}}\times pop_{05}^{\text{1}}\times 0.596=2\times 13\times 0.596=15.496$; $P_{05}^{\text{2}}\times pop_{05}^{\text{2}}\times (1-0.596)=3\times 16\times (1-0.596)=19.392$; $P_{53}^{\text{2}}\times pop_{53}^{\text{2}}\times 0.45=1\times 24\times 0.45=10.8$; $P_{53}^{\text{3}}\times pop_{53}^{\text{3}}\times (1-0.45)=2\times 25\times (1-0.45)=27.5$, resulting in a risk of 15.496+19.392+10.8+27.5=73.188 and a cost of 33.54+14.32+27.66=75.72.

The path for vehicle k=3 is $v_{\text{0}}\to v_{\text{4}}\to v_{\text{6}}\to v_{\text{0}}$, where it waits for 8 minutes at customer 4, transitioning into time period 3. The risk calculation is as follows: $P_{04}^{\text{1}}\times pop_{04}^{\text{1}}\times 0.625=1\times 24\times 0.625=15$; $P_{04}^{\text{2}}\times pop_{04}^{\text{2}}\times \text{(}1-0.625\text{)}=1.5\times 21\times \text{(}1-0.625\text{)}=11.813$; $P_{46}^{\text{3}}\times pop_{46}^{\text{3}}=2\times 30=60$, resulting in a risk of 15+11.813+60=86.813 and a cost of 32.02+11.18+36.4=79.6.

In summary, the total cost is represented by objective function 1, $f_{\text{1}}=58.58+75.72+79.6=239.318$, and the total risk is represented by objective function 2, $f_{\text{2}}=86.53+73.188+86.813=246.531$.

Encode the above path as [1,2,5,3,4,6,0,0,0,18,0,0,8,0,0].

(2) Weight vector initialization

Let the number of subproblems be N, and each subproblem is assigned a weight vector $\lambda^{h}=\left(\lambda_{\text{1}}^{h},\lambda_{\text{2}}^{h}\right)$, such that $\lambda_{\text{1}}^{h}+\lambda_{\text{2}}^{h}=1$. All weight vectors form a set $W_{v}=(\lambda^{\text{1}},\lambda^{\text{2}},...,\lambda^{N})$, which are uniformly distributed in the objective space and used for scalarization of the multi-objective function. During the initialization process, each subproblem corresponds to a solution $x^{h}$, forming the initial population $IP=(x^{\text{1}},x^{\text{2}},...,x^{N})$. To construct the neighborhood structure, the Euclidean distance between the weight vector of each subproblem h and the other weight vectors is computed. The closest T subproblems are selected to form the neighborhood set B(h). The scalarized objective function is represented by a weighted sum method. For subproblem h, its scalarized objective function is defined as $g^{h}\left(x\right)=\sum_{i=1}^{m}\lambda_{i}^{h}f_{i}\left(x\right)$, where m=2 represents the number of objectives, corresponding to the total transportation cost $f_{\text{1}}\left(x\right)$ and the total transportation risk $f_{\text{2}}\left(x\right)$, and $\lambda_{i}^{h}$ is the weight coefficient for the i -th objective of subproblem h. This scalar function will serve as the basis for comparing the solution quality during the neighborhood update phase.

Below is an example of this step. Suppose the number of subproblems is N=5, and the following weight vectors are generated: $\lambda^{\text{1}}=\left(1,0\right)$, $\lambda^{\text{2}}=(0.25,0\mathrm{.75})$, $\lambda^{\text{3}}=(0.5,0\mathrm{.5})$, $\lambda^{\text{4}}=(0.75,0\mathrm{.25})$, $\lambda^{\text{5}}=\left(0,1\right)$. Let the neighborhood size be T=3, so the neighborhood set for each subproblem is $B\left(1\right)=\left\{\lambda^{\text{1}},\lambda^{\text{2}},\lambda^{\text{3}}\right\}$, $B\left(2\right)=\left\{\lambda^{\text{1}},\lambda^{\text{2}},\lambda^{\text{3}}\right\}$, $B\left(3\right)=\left\{\lambda^{\text{2}},\lambda^{\text{3}},\lambda^{\text{4}}\right\}$, $B\left(4\right)=\left\{\lambda^{\text{3}},\lambda^{\text{4}},\lambda^{\text{5}}\right\}$, $B\left(5\right)=\left\{\lambda^{\text{3}},\lambda^{\text{4}},\lambda^{\text{5}}\right\}$. By selecting the weight vector $\lambda^{\text{3}}=(0.5,0\mathrm{.5})$, the initial solution’s scalarized objective function value is $g^{\left(3\right)}\left(x\right)=\lambda_{\text{3}}^{\text{1}}\times f_{\text{1}}+\lambda_{\text{3}}^{\text{2}}\times f_{\text{2}}=0.5\times 239.318+0.5\times 246.531=242.925$.

#### Evolutionary operation stage.

(1) **Dynamic selection of crossover and mutation operators based on PPO**
  1) **PPO-improved crossover and mutation strategy design**

Crossover and mutation are critical operations for generating new solutions in the MOEA/D algorithm. Crossover generates new candidate solutions by combining information from neighboring solutions, while mutation introduces diversity to the solutions, preventing the population from getting stuck in local optima. In traditional MOEA/D algorithms, crossover and mutation typically follow fixed rules, such as random crossover and uniform mutation. To further enhance the evolutionary efficiency of MOEA/D, PPO is employed during the crossover and mutation stages. The PPO strategy dynamically selects a combination of crossover and mutation operators based on the population state, replacing the traditional fixed operators.

The state space includes the evolutionary progress $evo_{p}$, the population diversity indicator $div_{p}$, the stagnation flag due to lack of improvement $sta_{f}$, the similarity between the two parents $sim_{p}$, the quality difference between the parents $diff_{p}$, and the frequency of current operator usage $cur_{ou}$. The complete state space is represented by $S_{t}=\left\{evo_{p},div_{p},sta_{f},sim_{p},diff_{p},cur_{ou}\right\}$. The action space includes the selection of crossover operators $Cro_{o}$ and mutation operators $Mut_{o}$. The crossover operator selection $Cro_{o}$ is given by $Cro_{o}=\left\{o_{c1},o_{c2},o_{c3},o_{c4}\right\}$, corresponding to OX crossover, PMX crossover, cycle crossover, and path-based crossover. The mutation operator selection $Mut_{o}$ is given by $Mut_{o}=\left\{o_{m1},o_{m2},o_{m3},o_{m4}\right\}$, corresponding to 2-opt mutation, Relocate mutation, Swap mutation, and Inversion mutation. The action space is the combination of crossover and mutation operators, denoted as $act_{t}=\left\{(o_{ci},o_{mj})|i=1,...,4;j=1,...,4\right\}$. The reward function includes the quality improvement reward $rew_{q}$, the diversity reward $rew_{d}$, the population stagnation breakthrough reward $rew_{p}$, the stage adaptability reward $rew_{s}$, and the constraint violation penalty $cv_{p}$. The complete reward function is denoted as $Rew_{t}=\left\{rew_{q},rew_{d},rew_{p},rew_{s},cv_{p}\right\}$.

The following are the steps for the crossover and mutation stage of the PPO-MOEA/D algorithm.

**Table:** 

Algorithm 2: PPO Algorithm-Enhanced MOEA/D Crossover and Mutation
Step 1. Initialization. Randomly generate an initial population of solutions, where each solution represents a path plan. Generate a set of weight vectors $A=\left\{\lambda^{\text{1}},\lambda^{\text{2}},...,\lambda^{N}\right\}$, where $\lambda_{i}^{\left(m\right)}\in \left[0,1\right]$ and $\sum_{m=1}^{M}\lambda_{i}^{\left(m\right)}=1$. Define the neighborhood set for each weight vector based on the Euclidean distance between the weight vectors. Initialize the policy parameters θ, value network parameters ϕ. Set the initial values for crossover probability $cp_{iv}$, mutation probability $mp_{iv}$, and mutation amplitude $amp_{m}$.
Step 2. Iterative Optimization. Each weight vector is associated with a subproblem. The objective is to minimize a weighted decomposition objective function: $F_{i}\left(z|\lambda_{i}\right)=\underset{1\leq m\leq M}{max}\left\{\lambda_{i}^{\left(m\right)}|f_{m}\left(z\right)-z_{m}\right\}$, where $f_{m}\left(z\right)$ is the objective function and $z_{m}$ is the ideal value of the objective in the current population.
Step 3. PPO Dynamic Operator Selection. The PPO policy network receives state vector $S_{t}$ (containing population diversity, evolution stage, historical operator performance, etc.) and outputs action probability distribution $\pi_{\theta }\left(S_{t}\right)$.
Step 4. Execute Dynamic Operator Combination. Execute evolutionary operations according to the PPO-selected operator combination.
Step 5. Reward Calculation. Calculate the reward function $Rew_{t}$. Step 6. Based on the reward function, update the PPO policy network parameters and the value network parameters to optimize the policy network.

2) **PPO-improved crossover and mutation operator selection**

Let the current subproblem index be h=3, and its corresponding neighborhood be B(3). Two parent solutions $x_{a}$ and $x_{b}$ are randomly selected from B(3). For this step, we select solutions $x_{\text{2}}$ and $x_{\text{4}}$ from $B\left(3\right)=\left\{\lambda^{\text{2}},\lambda^{\text{3}},\lambda^{\text{4}}\right\}$, where $x_{\text{2}}$ is one of the initial solutions chosen as a parent solution, denoted as $x_{a}$, and $x_{\text{4}}$ is the other solution in the neighborhood, denoted as $x_{b}$. PPO analyzes that the current population has low diversity, so it selects the “OX crossover operator combined with Relocate mutation operator” to enhance exploration. From the population, the selected parent $x_{b}=\left[2,3,1,6,4,5,0,0,8,0,0,0,0,0,0\right]$ and parent $x_{a}=\left[1,2,5,3,4,6,0,0,0,18,0,0,8,0,0\right]$ are chosen. First, an OX crossover is applied to the customer node order portion, resulting in a child customer node sequence of [2,1,5,3,4,6]. Then, a Relocate mutation is applied to the child path, relocating customer 1 to position 4, yielding the mutated path [2,5,3,1,4,6]. The time-varying decoder is then invoked, and the departure time and waiting time are optimized based on the mutated path. The complete encoded value is [2,5,3,1,4,6,0,0,0,0,0,13,8,0,0].

The path for vehicle k=1 is $v_{\text{0}}\to v_{\text{2}}\to v_{\text{5}}\to v_{\text{0}}$, with no waiting throughout the entire route. The risk calculation is as follows: $P_{02}^{\text{1}}\times pop_{02}^{\text{1}}\times 0.686=2.5\times 25\times 0.686=42.875$; $P_{02}^{\text{2}}\times pop_{02}^{\text{2}}\times \text{(}1-0.686\text{)}=3\times 18\times \text{(}1-0.686\text{)}=16.956$; $P_{25}^{\text{2}}\times pop_{25}^{\text{2}}\times 0.6=1\times 12\times 0.6=7.2$,$P_{25}^{\text{3}}\times pop_{25}^{\text{3}}\times (1-0.6)=1\times 12\times (1-0.6)=7.2$, resulting in a risk of 42.875+16.956+7.2+7.2=74.231 and a cost of 29.15+18.03+33.54=80.72.

The path for vehicle k=2 is $v_{\text{0}}\to v_{\text{5}}\to v_{\text{3}}\to v_{\text{0}}$, where it waits for 13 minutes at customer 3, transitioning into time period 3. The risk calculation is as follows: $P_{03}^{\text{1}}\times pop_{03}^{\text{1}}\times 0.723=1.5\times 18\times 0.723=19.521$; $P_{03}^{\text{2}}\times pop_{03}^{\text{2}}\times (1-0.723)=1\times 13\times (1-0.723)=3.601$; $P_{31}^{\text{3}}\times pop_{31}^{\text{3}}=3.5\times 6=21$, resulting in a risk of 19.521+3.601+21=44.122 and a cost of 27.66+8.06+22.36=58.08.

The path for vehicle k=3 is $v_{\text{0}}\to v_{\text{4}}\to v_{\text{6}}\to v_{\text{0}}$, where it waits for 8 minutes at customer 4, transitioning into time period 3. The risk calculation is as follows: $P_{04}^{\text{1}}\times pop_{04}^{\text{1}}\times 0.625=1\times 24\times 0.625=15$; $P_{04}^{\text{2}}\times pop_{04}^{\text{2}}\times \text{(}1-0.625\text{)}=1.5\times 21\times \text{(}1-0.625\text{)}=11.813$; $P_{46}^{\text{3}}\times pop_{46}^{\text{3}}=2\times 30=60$, resulting in a risk of 15+11.813+60=86.813 and a cost of 32.02+11.18+36.4=79.6.

In summary, the total cost is represented by objective function 1, $f_{\text{1}}=80.72+58.08+79.6=218.4$, and the total risk is represented by objective function 2, $f_{\text{2}}=74.231+44.122+86.813=205.166$. After scalarization, the objective function value is $g^{\left(3\right)}\left(x\right)=\lambda_{\text{3}}^{\text{1}}\times f_{\text{1}}+\lambda_{\text{3}}^{\text{2}}\times f_{\text{2}}=0.5\times 218.4+0.5\times 205.166=211.783$, which is superior to the initial solution of 242.925. The initial solution of 242.925 is discarded, and the solution obtained through crossover and mutation is retained.

The parent paths are executed using the combination of the OX crossover operator and the Relocate mutation operator, as shown in Figs 6 and 7.

**Fig 6:**
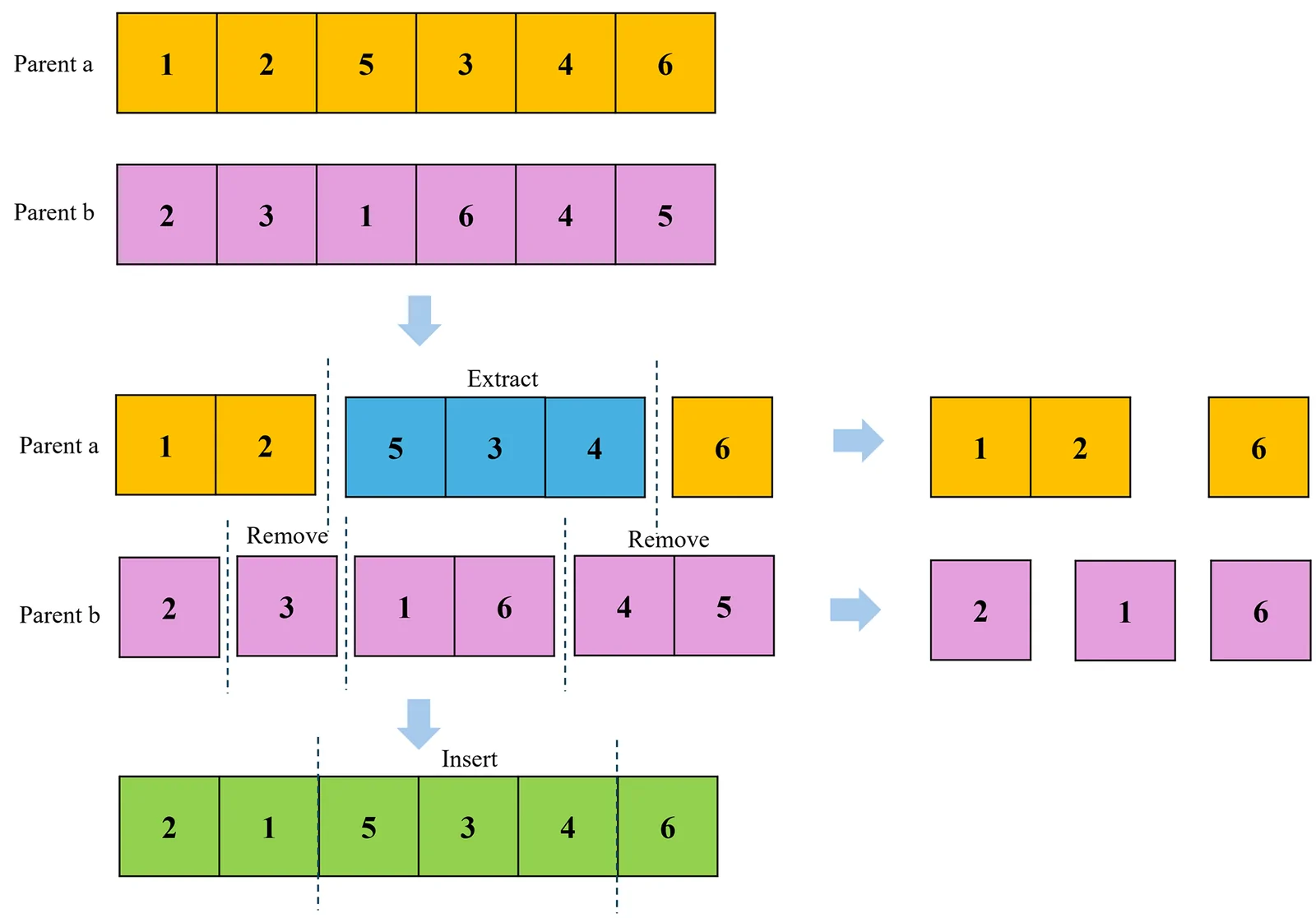
Example of ox operator.

**Fig 7:**
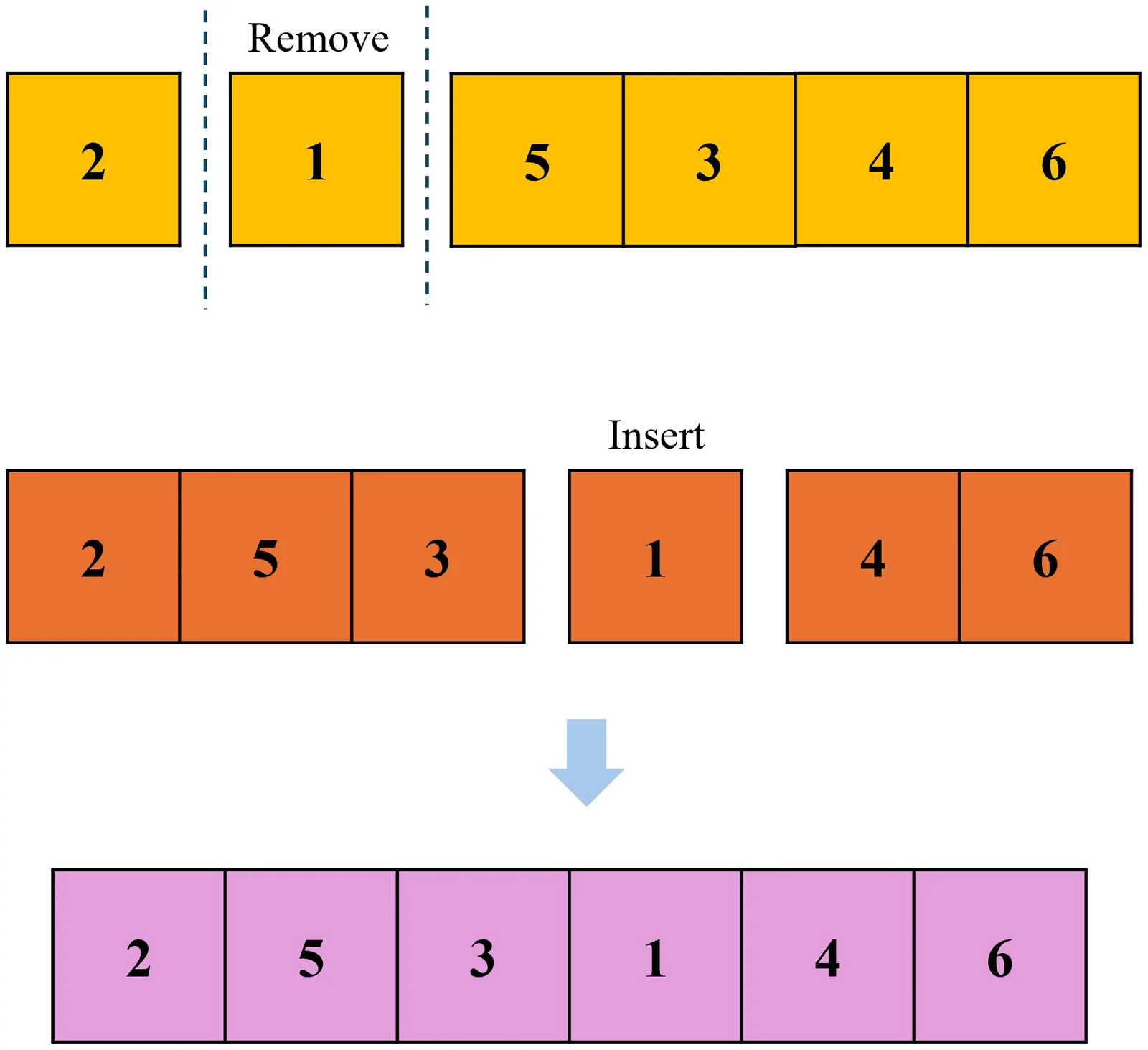
Example of relocate operator.

(2) **Local search optimization**

After the offspring solutions are optimized using the PPO strategy, this stage no longer utilizes the PPO network. Instead, it follows the neighborhood search process in the traditional MOEA/D method, using a destruction-repair operator, as shown in Fig 8. After each operation, the scalarized objective function value is calculated. If the new solution is better than the current solution, it is accepted, and the current individual is updated. If no improvement is found, the search moves to the next neighborhood structure. The destruction-repair operator is executed by removing customer nodes 3 and 4 from the path. During the repair phase, customer node 3 is inserted at position 3, resulting in the path [2,5,1,3,6]. Customer node 4 is then inserted at position 2, and the reassessing through local simulaepaired path becomes [2,5,4,1,3,6].

**Fig 8:**
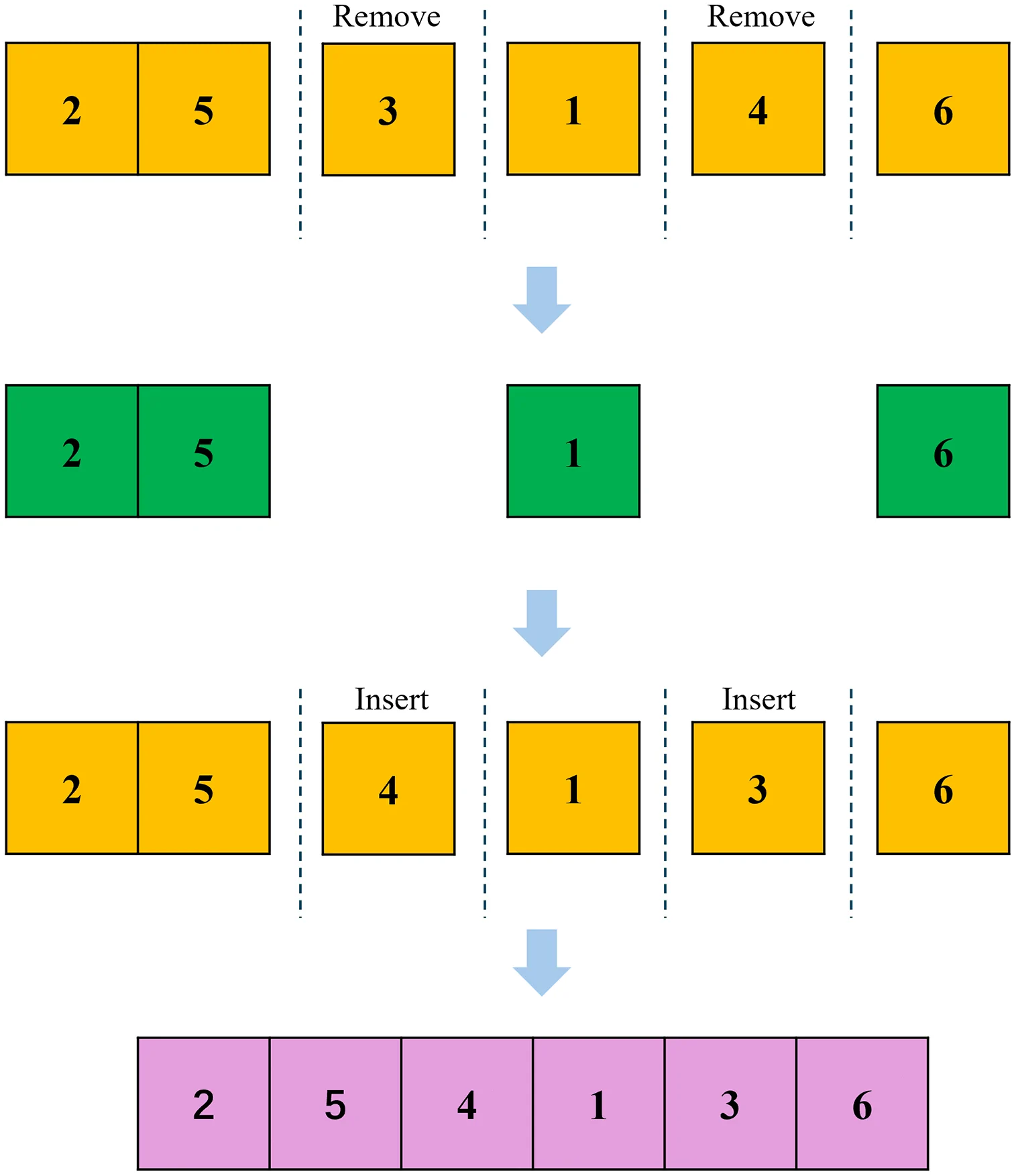
Destruction-repair operator.

The path for vehicle k=1 is $v_{\text{0}}\to v_{\text{2}}\to v_{\text{5}}\to v_{\text{0}}$, with no waiting throughout the entire route. The risk calculation is as follows: $P_{02}^{\text{1}}\times pop_{02}^{\text{1}}\times 0.686=2.5\times 25\times 0.686=42.875$; $P_{02}^{\text{2}}\times pop_{02}^{\text{2}}\times \text{(}1-0.686\text{)}=3\times 18\times \text{(}1-0.686\text{)}=16.956$; $P_{25}^{\text{2}}\times pop_{25}^{\text{2}}\times 0.6=1\times 12\times 0.6=7.2$,$P_{25}^{\text{3}}\times pop_{25}^{\text{3}}\times (1-0.6)=1\times 12\times (1-0.6)=7.2$, resulting in a risk of 42.875+16.956+7.2+7.2=74.231 and a cost of 29.15+18.03+33.54=80.72.

The path for vehicle k=2 is $v_{\text{0}}\to v_{\text{4}}\to v_{\text{1}}\to v_{\text{0}}$, where it waits for 8 minutes at customer 4, transitioning into time period 3. The risk calculation is as follows: $P_{04}^{\text{1}}\times pop_{04}^{\text{1}}\times 0.624=1\times 24\times 0.624=14.976$; $P_{04}^{\text{2}}\times pop_{04}^{\text{2}}\times (1-0.624)=1.5\times 21\times (1-0.624)=11.844$; $P_{41}^{\text{2}}\times pop_{41}^{\text{2}}=1\times 3=3$, resulting in a risk of 14.976+11.844+3=29.82 and a cost of 32.02+15+22.36=69.38.

The path for vehicle k=3 is $v_{\text{0}}\to v_{\text{3}}\to v_{\text{6}}\to v_{\text{0}}$, where it waits for 12.34 minutes at the distribution center. The risk calculation is as follows: $P_{03}^{\text{1}}\times pop_{03}^{\text{1}}\times 0.72=1.5\times 18\times 0.72=7.56$; $P_{03}^{\text{2}}\times pop_{03}^{\text{2}}\times \text{(}1-0.28\text{)}=1\times 13\times \text{(}1-0.28\text{)}=9.36$; $P_{36}^{\text{3}}\times pop_{36}^{\text{3}}=1\times 7=7$, resulting in a risk of 7.56+9.36+7=23.92 and a cost of 27.66+19.72+36.4=83.78.

In summary, the total cost is represented by objective function 1, $f_{\text{1}}=80.72+69.38+83.78=233.88$, and the total risk is represented by objective function 2, $f_{\text{2}}=74.231+29.82+23.92=127.971$. After scalarization, the objective function value is $g^{\left(3\right)}\left(x\right)=\lambda_{\text{3}}^{\text{1}}\times f_{\text{1}}+\lambda_{\text{3}}^{\text{2}}\times f_{\text{2}}=0.5\times 233.88+0.5\times 127.971=180.926$, which is non-dominated by the solution obtained through crossover and mutation, and is therefore retained.

#### Update and termination stage.

(1) **Neighborhood solution update**

After completing the local search and obtaining a new optimized solution, it is necessary to update the current solution based on the fundamental principles of the MOEA/D algorithm. For each subproblem h, there is a neighborhood solution set B(h). The current new solution $g^{new}$ will be compared with the current optimal solutions of all subproblems in the neighborhood. If the objective value of $g^{new}$ is better than that of any solution in the neighborhood, the original solution is replaced. For subproblem h=3, the neighborhood set is $B\left(3\right)=\left(\lambda^{\text{2}},\lambda^{\text{3}},\lambda^{\text{4}}\right)$. The objective function values corresponding to each direction vector are as follows: $x_{\text{2}}$ corresponds to $g^{\left(2\right)}=0.75\times 233.88+0.25\times 127.971=207.40275$, $x_{\text{3}}$ corresponds to $g^{\left(3\right)}=0.5\times 233.88+0.5\times 127.971=180.926$, and $x_{\text{4}}$ corresponds to $g^{\left(4\right)}=0.25\times 233.88+0.75\times 127.971=154.448$. After the update, the new solution becomes the common solution for subproblems 2, 3, and 4, with the corresponding aggregated values being $g^{new\left(2\right)}=207.40275$,$g^{new\left(3\right)}=180.926$,$g^{new\left(4\right)}=154.448$, respectively.

(2) **Non-dominated solution set update**

During the algorithm’s iteration process, to maintain the diversity and representativeness of the optimal solutions, the MOEA/D framework maintains a global non-dominated solution set to record the non-dominated solutions generated in each evolutionary cycle. Let the current solution 1 be $f_{\text{1}}=218.4,f_{\text{2}}=205.166$ and solution 2 be $f_{\text{1}}=233.88,f_{\text{2}}=127.971$. Both solutions lie on the non-dominated front, and therefore, both are retained.

(3) **Termination conditions and output**

The PPO-MOEA/D multi-objective optimization algorithm in this study terminates upon satisfying any of the following conditions: reaching the predefined maximum number of iterations, or when the external non-dominated solution set (i.e., the Pareto optimal solution set) remains stable over several consecutive generations, indicating that the population has converged. Once the termination conditions are met, the algorithm outputs the final non-dominated solution set, providing decision-makers with a variety of optimized solutions.

The detailed flowchart of PPO-MOEA/D is shown in Fig 9.

**Fig 9:**
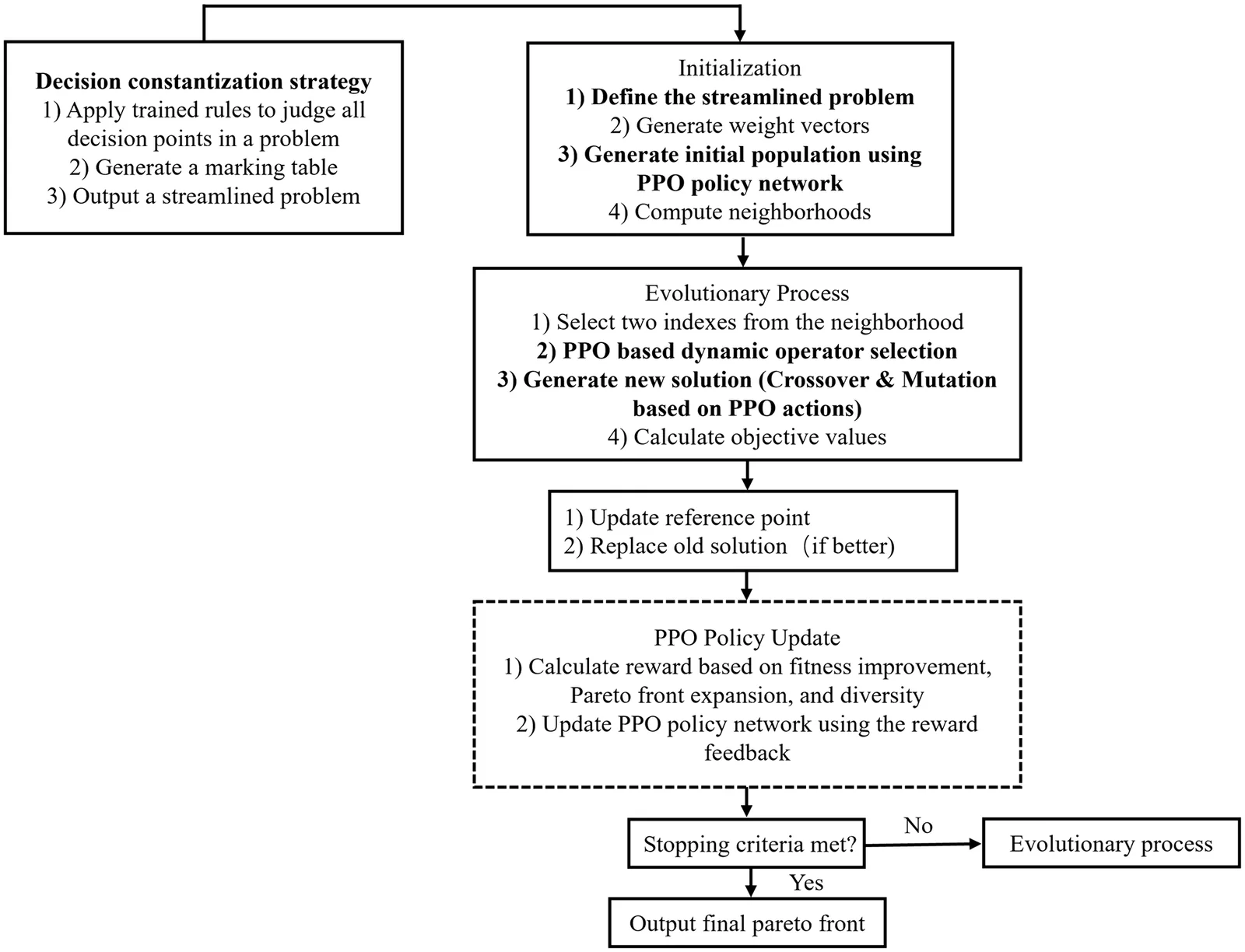
PPO-MOEA/D flowchart.

## Numerical analysis

In this section, we will test the models and algorithms described above. The proposed method is implemented in Python 3.8 and integrates Gurobi (version 11.0). All experiments are conducted on a PC equipped with a 4.0 GHz multi-core processor and 32GB of RAM, running Windows 11, with execution performed using PyCharm.

Four instances of different scales are randomly generated, with each scale consisting of 6 groups. The first scale consists of instances containing one distribution center and 9 customers, labeled as datasets data1-9c to data6-9c. The second scale contains one distribution center and 49 customers, labeled as datasets data1-49c to data6-49c. The third scale includes one distribution center and 74 customers, labeled as datasets data1-74c to data6-74c. The fourth scale consists of one distribution center and 99 customers, labeled as datasets data1-99c to data6-99c. The distribution center and all customers are randomly placed in a square plane, with coordinates for all points randomly generated within the range [0,50]. The distribution center is fixed at coordinates (50,50), and the distance between nodes is represented using Euclidean distance between vertices, satisfying the triangle inequality. The accident rates for each edge in each time period are randomly generated within the range [1%, 6%], and the population on each edge is randomly generated within the range [1, 100]. There are 24 time periods, each lasting 20 minutes, with the assumption that the vehicle speed on each edge is 1. Since the unit transportation cost c is a fixed parameter, it is set to c=1. The vehicle load capacity is set to Q=30, and the customer demand is randomly generated within the range [1, 30], ensuring that $q_{i}$ does not exceed Q. The instance generation method for these four scales is consistent, and the example problems used in the numerical experiments are shown in Table 8.

**Table 8:** Test instances for numerical experiments.

Index	Instance	Number of Nodes	Vehicle Load Capacity
1	data1-9c	10	30
2	data2-9c	10	30
3	data3-9c	10	30
4	data4-9c	10	30
5	data5-9c	10	30
6	data6-9c	10	30
7	data1-49c	50	30
8	data2-49c	50	30
9	data3-49c	50	30
10	data4-49c	50	30
11	data5-49c	50	30
12	data6-49c	50	30
13	data1-74c	75	30
14	data2-74c	75	30
15	data3-74c	75	30
16	data4-74c	75	30
17	data5-74c	75	30
18	data6-74c	75	30
19	data1-99c	100	30
20	data2-99c	100	30
21	data3-99c	100	30
22	data4-99c	100	30
23	data5-99c	100	30
24	data6-99c	100	30

### Validation of the preprocessing strategy

To validate whether the proposed node departure-time decision preprocessing strategy erroneously removes potentially important waiting decisions, we run both the uncompressed full model and the preprocessed compressed model on the 10-node instances. The solution quality and computation time of the two models are compared. Each instance is independently executed 10 times and the average values are reported. The results are shown in Table 9.

**Table 9:** Validation results of the preprocessing strategy.

Instance	HV (full model)	HV (preprocessed)	HV deviation	Time (full model) / s	Time (preprocessed) / s	Time saving rate
Date1-9c	0.8207	0.8298	+1.11%	126	95	24.6%
Date2-9c	0.7636	0.7529	−1.40%	129	103	20.2%
Date3-9c	0.8142	0.8251	+1.34%	177	141	20.3%
Date4-9c	0.7560	0.7478	−1.08%	147	118	19.7%
Date5-9c	0.9207	0.9316	+1.18%	120	79	34.2%
Date6-9c	0.8533	0.8648	+1.35%	133	99	25.6%

The results show that the HV deviations of the preprocessing strategy on all test instances do not exceed ±1.40%, indicating that the compressed decisions are all non-critical and no substantial loss in solution quality is incurred. Meanwhile, the preprocessing significantly reduces the dimensionality of the decision variables, achieving an average time saving of 24.1%. This verifies that the preprocessing strategy effectively reduces the search space while preserving solution quality, and does not carry the risk of erroneously removing important waiting decisions.

### Ablation study

To separately evaluate the contributions of the three core improvements in PPO-MOEA/D, we design five algorithm variants for comparative experiments: Υ1 is the traditional MOEA/D without any improvement; Υ2 adds only the preprocessing strategy to Υ1; Υ3 adds only the PPO-based initialization to Υ1; Υ4 adds only the PPO-based adaptive operator selection to Υ1; Υ5 is the complete PPO-MOEA/D incorporating all three components. Two representative instances are selected at each of the 10, 50, 75, and 100-node scales. Each instance is independently run 10 times, and the average values and standard deviations of HV and computation time are recorded. The results are shown in Table 10.

**Table 10:** Ablation study results.

Instance	Metric	Υ1	Υ2	Υ3	Υ4	Υ5
Data5-9c	HV	0.713 ± 0.013	0.791 ± 0.010	0.734 ± 0.011	0.842 ± 0.009	0.876 ± 0.008
	Time(s)	48 ± 3.9	42 ± 3.5	51 ± 4.1	55 ± 4.0	53 ± 3.6
Data6-9c	HV	0.495 ± 0.019	0.588 ± 0.014	0.756 ± 0.012	0.651 ± 0.015	0.863 ± 0.009
	Time(s)	56 ± 4.5	41 ± 3.7	59 ± 4.3	61 ± 4.6	48 ± 3.9
Data5-49c	HV	0.684 ± 0.021	0.728 ± 0.017	0.701 ± 0.016	0.793 ± 0.014	0.812 ± 0.012
	Time(s)	86 ± 6.2	71 ± 5.5	83 ± 6.0	91 ± 6.4	65 ± 5.2
Data6-49c	HV	0.532 ± 0.024	0.667 ± 0.018	0.599 ± 0.019	0.714 ± 0.016	0.788 ± 0.013
	Time(s)	98 ± 6.8	88 ± 5.9	103 ± 6.5	106 ± 6.9	109 ± 6.2
Data5-74c	HV	0.746 ± 0.025	0.813 ± 0.020	0.769 ± 0.021	0.792 ± 0.018	0.884 ± 0.014
	Time(s)	103 ± 7.1	87 ± 6.4	107 ± 7.3	111 ± 6.9	100 ± 6.6
Data6-74c	HV	0.564 ± 0.026	0.625 ± 0.021	0.708 ± 0.020	0.743 ± 0.018	0.902 ± 0.015
	Time(s)	128 ± 7.8	109 ± 6.9	126 ± 7.5	131 ± 7.3	117 ± 6.8
Data5-99c	HV	0.591 ± 0.029	0.665 ± 0.023	0.723 ± 0.021	0.751 ± 0.019	0.867 ± 0.014
	Time(s)	118 ± 7.5	105 ± 6.7	124 ± 7.3	130 ± 7.0	134 ± 6.8
Data6-99c	HV	0.453 ± 0.031	0.548 ± 0.024	0.672 ± 0.022	0.714 ± 0.020	0.811 ± 0.017
	Time(s)	134 ± 8.0	121 ± 7.4	138 ± 7.8	142 ± 7.6	111 ± 6.9

Based on the results in Table 10, the following conclusions can be drawn. Each individual improvement component contributes significantly. The HV values of Υ2, Υ3, and Υ4 are all higher than that of the baseline Υ1, indicating that the preprocessing strategy, PPO-based initialization, and PPO-based adaptive operator selection each effectively improves algorithmic performance. Among them, PPO-based initialization primarily contributes to enhancing convergence quality; the preprocessing strategy reduces computation time in most instances; and PPO-based operator selection demonstrates more prominent advantages on complex large-scale instances. A synergistic effect exists among the three improvement components. The Υ5 achieves higher HV values than any single-component variant on all instances, and the improvement exceeds the sum of individual contributions. Taking data6-99c as an example, the HV improvements of Υ2, Υ3, and Υ4 over Υ1 are 0.095, 0.219, and 0.261, respectively, summing to 0.575, while the total improvement of Υ5 over Υ1 is 0.358, indicating positive interactions among the components rather than a simple additive effect.

The unique value of the preprocessing strategy is also observed. Compared with Υ1, Υ2 achieves improvements in HV values while simultaneously reducing computation time across all instances, further verifying that the preprocessing strategy effectively reduces the search space by eliminating non-critical waiting decisions. Regarding the variation trend of each component with problem scale, on small-scale instances, the contributions of the three components are comparable. As the problem scale increases to 100 nodes, the contribution of each component grows significantly, with PPO-based operator selection showing the most pronounced increase, indicating that the adaptive operator selection mechanism is more advantageous in complex search spaces.

### Performance testing of MOEA/D, NSGA-Ⅱ and PPO-MOEA/D algorithms

This section provides a comparative analysis of the performance of PPO-MOEA/D against the conventional MOEA/D and NSGA-Ⅱ algorithm. Initially, we compare the objective values achieved by PPO-MOEA/D, the traditional MOEA/D and NSGA-Ⅱ across 24 distinct sets of randomly generated instances. Subsequently, the computational times and HV of both algorithms are contrasted. Each dataset is executed 10 times, and the average value is computed. Figs 10–13 illustrates the objective values of the solutions derived from PPO-MOEA/D, traditional MOEA/D and NSGA-Ⅱ for four different dataset scales.

**Fig 10:**
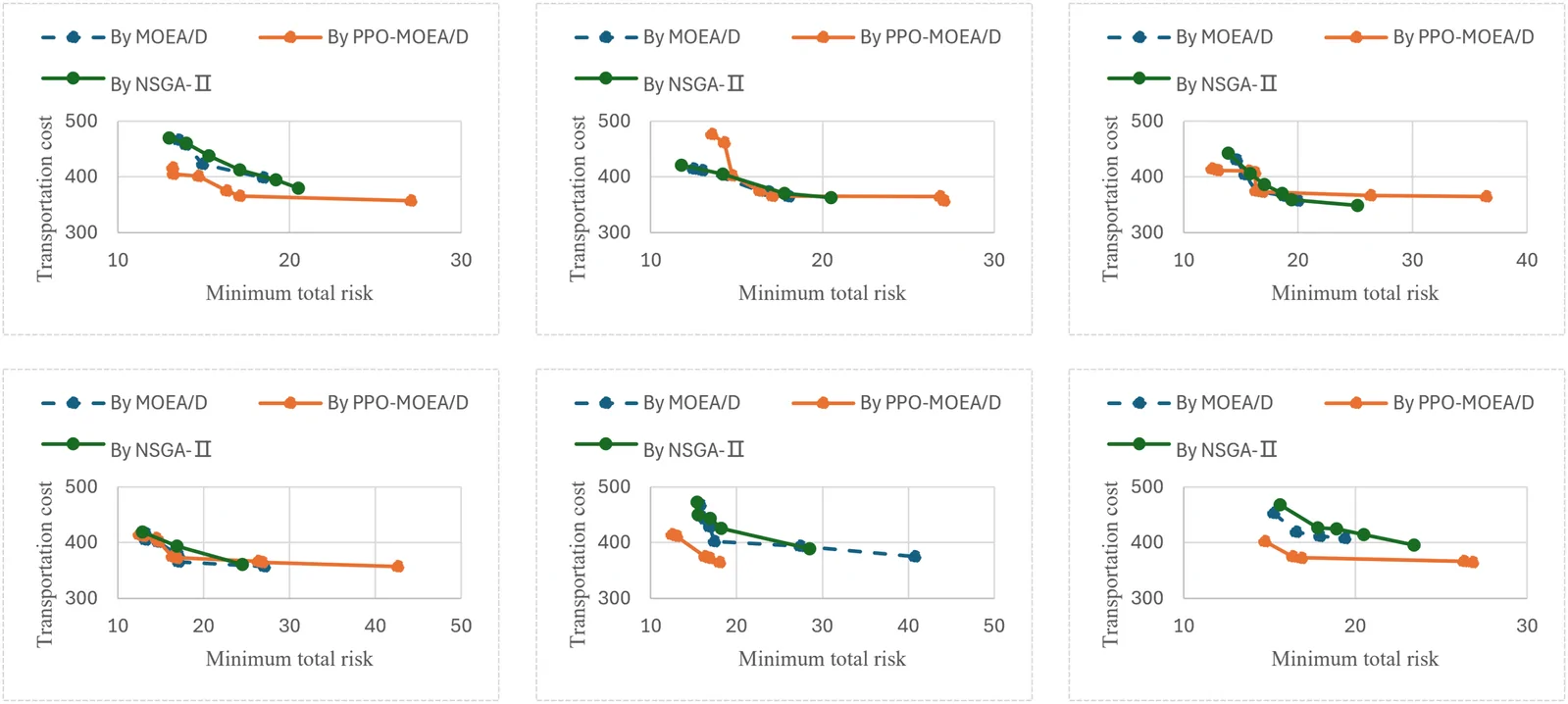
Pareto fronts of MOEA/D, NSGA-Ⅱ and PPO-MOEA/D for the 10-node instance.

**Fig 11:**
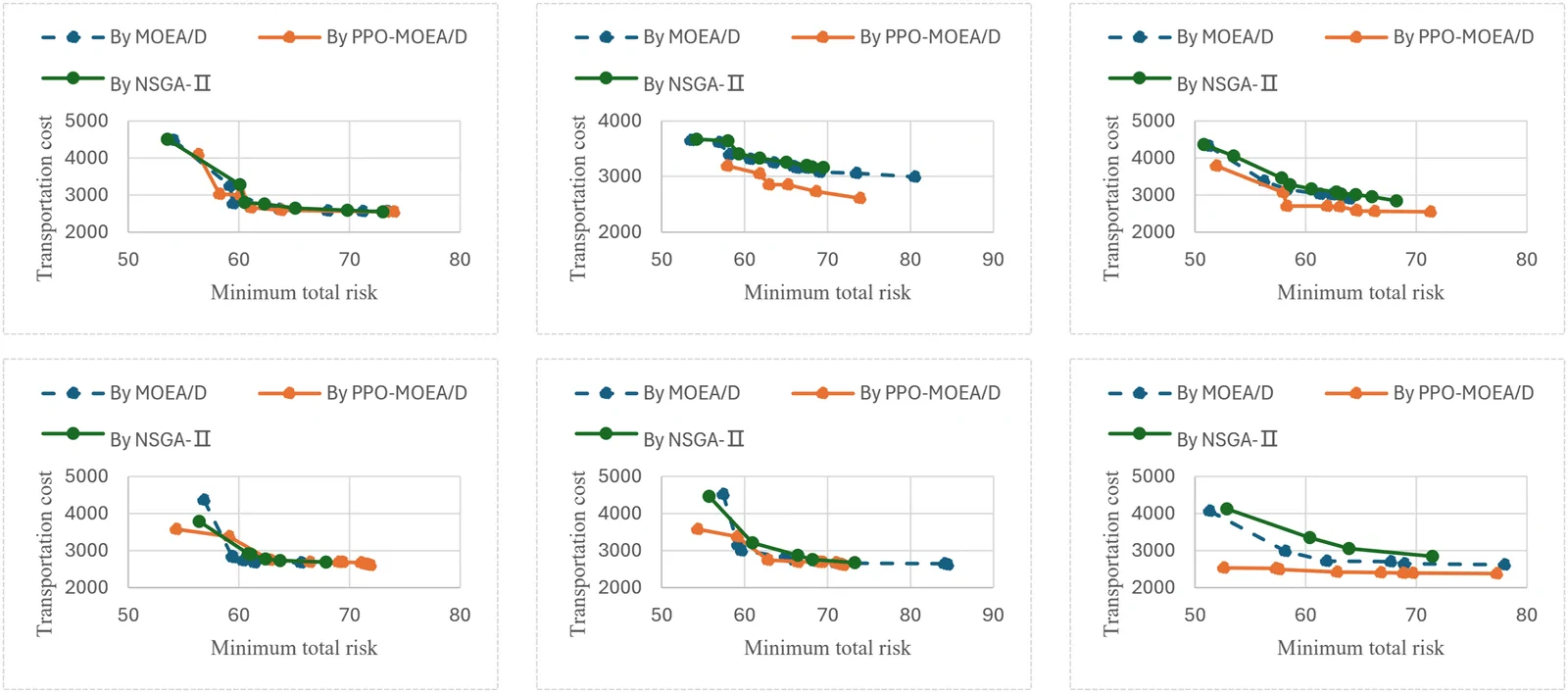
Pareto fronts of MOEA/D, NSGA-Ⅱ and PPO-MOEA/D for the 50-node instance.

**Fig 12:**
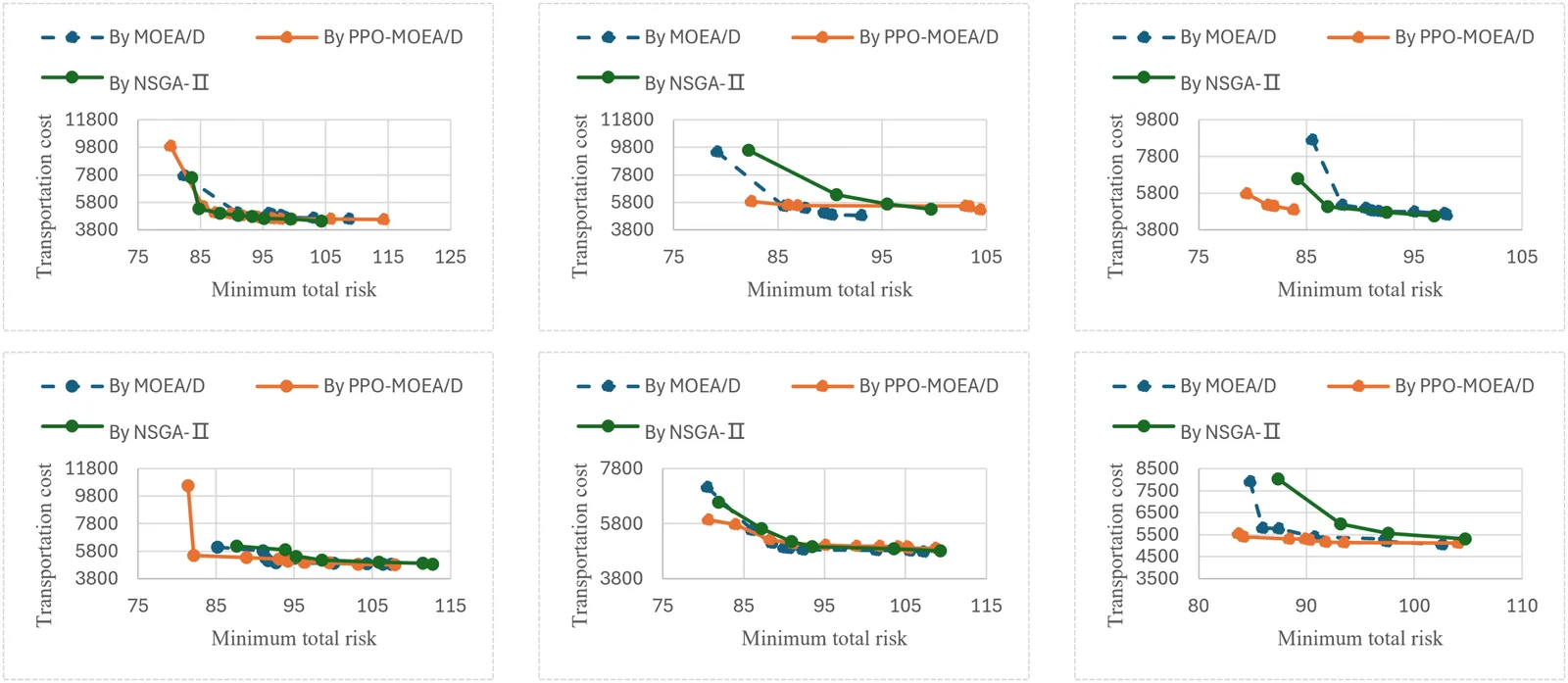
Pareto fronts of MOEA/D, NSGA-Ⅱ and PPO-MOEA/D for the 75-node instance.

**Fig 13:**
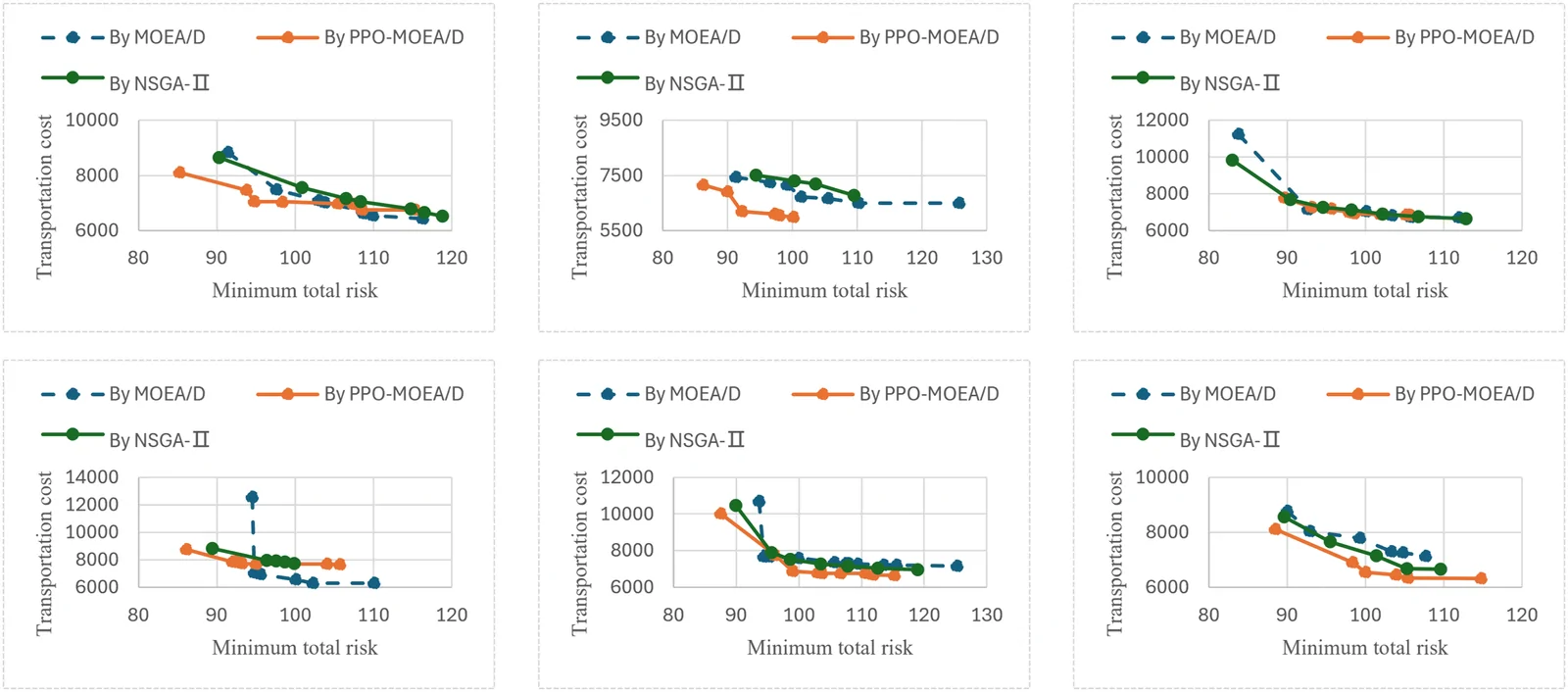
Pareto fronts of MOEA/D, NSGA-Ⅱ and PPO-MOEA/D for the 100-node instance.

A comparative analysis of the 24 sets of Pareto front solutions obtained by MOEA/D, NSGA-II, and PPO-MOEA/D across different node scales clearly highlights the performance disparities among the three algorithms. While all three algorithms exhibit strong optimization capabilities, PPO-MOEA/D consistently outperforms in most instances, particularly in achieving an optimal balance between total cost and total risk. For the 10-node problem, the performance of the three algorithms is relatively similar, yet PPO-MOEA/D demonstrates its superiority in managing multiple objectives simultaneously. In the case of data1-9c, the total cost and total risk of MOEA/D and NSGA-II closely approximate those of PPO-MOEA/D; however, PPO-MOEA/D, leveraging its reinforcement learning mechanism, is able to generate a broader range of solutions, notably maintaining lower costs in high-risk areas. As the problem size increases, the advantages of PPO-MOEA/D become even more pronounced. In the 50-node problem, PPO-MOEA/D consistently yields a significantly lower total cost at reduced risk levels, as demonstrated by data3-49c. For the 75-node and 100-node problems, the superiority of PPO-MOEA/D becomes increasingly evident, while NSGA-II outperforms MOEA/D on some large-scale instances but still falls short of PPO-MOEA/D. In conclusion, PPO-MOEA/D exhibits a clear advantage in the majority of cases, whereas MOEA/D and NSGA-II demonstrates relatively stable performance across all scales but is generally inferior to PPO-MOEA/D. By dynamically adjusting evolutionary operations via its reinforcement learning-based strategy network, PPO-MOEA/D significantly broadens the exploration scope within the objective space, while enhancing the boundary quality of the solution set.

The computation times shown in Table 11 are average values rounded to the nearest integer, each obtained from 10 independent runs. The results indicate that, for all 24 instances across the four scales, the computation times of all three heuristic algorithms are significantly less than that of the MILP method, demonstrating that the approximate algorithms effectively accelerate the solving process. Meanwhile, the computation time of NSGA-II is comparable to that of MOEA/D and PPO-MOEA/D.

**Table 11:** Comparison of computation times between MOEA/D, PPO-MOEA/D, and NSGA-II.

Group	Computation times by MOEA/D (s)	Computation times by PPO-MOEA/D (s)	Computation times by NSGA-II (s)	Computation times by MILP approach (low bound) (s)
Level 1-scale				
data 1-9c	55	65	62	149
data 2-9c	46	62	63	121
data 3-9c	48	53	55	194
data 4-9c	43	52	41	153
data 5-9c	31	48	46	134
data 6-9c	56	48	67	173
Level 2-scale				
data 1-49c	86	65	88	3600
data 2-49c	69	90	58	3600
data 3-49c	98	109	106	3600
data 4-49c	57	64	69	3600
data 5-49c	71	98	63	3600
data 6-49c	86	65	72	3600
Level 3-scale				
data 1-74c	103	100	105	3600
data 2-74c	106	123	111	3600
data 3-74c	128	117	115	3600
data 4-74c	117	103	130	3600
data 5-74c	111	113	109	3600
data 6-74c	137	123	136	3600
Level 4-scale				
data 1-99c	138	111	144	3600
data 2-99c	128	132	107	3600
data 3-99c	117	133	110	3600
data 4-99c	118	134	126	3600
data 5-99c	123	110	127	3600
data 6-99c	134	111	145	3600

The hypervolume (HV) metric is used to evaluate the convergence and diversity of the final solution set for each algorithm. As shown in Table 12, for the Level 1-scale (10-node) instances, PPO-MOEA/D achieves higher HV values than both MOEA/D and NSGA-II in four out of six cases, while being slightly lower in the remaining two cases. In the Level 2-scale (50-node) instances, PPO-MOEA/D outperforms both MOEA/D and NSGA-II in all six cases. In the Level 3-scale (75-node) instances, PPO-MOEA/D also achieves higher HV values than both benchmark algorithms across all instances, with a particularly significant improvement on data3-74c. In the Level 4-scale (100-node) instances, PPO-MOEA/D again outperforms both MOEA/D and NSGA-II in all six cases. Overall, PPO-MOEA/D significantly improves the algorithm’s overall performance across all scales, and its advantage becomes more pronounced as the problem size increases.

**Table 12:** Comparison of HVs between MOEA/D, PPO-MOEA/D and NSGA-II.

Group	HV			Rate of change of PPO-MOEA/D relative to the other two algorithms	
	MOEA/D	NSGA-II	PPO-MOEA/D	MOEA/D	NSGA-II
Level 1-scale					
data 1-9c	0.4922	0.7140	0.8412	41.49%	15.12%
data 2-9c	0.8265	0.7112	0.7523	−9.86%	5.46%
data 3-9c	0.7403	0.7503	0.8256	10.33%	9.12%
data 4-9c	0.8301	0.7244	0.7427	−11.77%	2.46%
data 5-9c	0.5789	0.6559	0.9311	37.83%	29.56%
data 6-9c	0.4087	0.6511	0.8634	52.66%	32.61%
Level 2-scale					
data 1-49c	0.7054	0.6973	0.7590	7.06%	8.13%
data 2-49c	0.3869	0.6412	0.6661	41.92%	3.74%
data 3-49c	0.5438	0.6638	0.7167	24.12%	7.38%
data 4-49c	0.6731	0.6551	0.7233	6.94%	9.43%
data 5-49c	0.7698	0.7276	0.8515	9.59%	14.55%
data 6-49c	0.5852	0.7755	0.9126	35.88%	15.02%
Level 3-scale					
data 1-74c	0.7595	0.7480	0.8155	6.87%	8.28%
data 2-74c	0.7223	0.7015	0.7866	8.17%	10.82%
data 3-74c	0.4871	0.6043	0.9021	46.00%	33.01%
data 4-74c	0.7914	0.6718	0.9142	13.43%	26.51%
data 5-74c	0.7478	0.7502	0.7945	5.88%	5.58%
data 6-74c	0.7784	0.6673	0.9377	16.99%	28.84%
Level 4-scale					
data 1-99c	0.4688	0.5217	0.6525	28.15%	20.05%
data 2-99c	0.3941	0.5763	0.8675	54.57%	33.57%
data 3-99c	0.6446	0.6334	0.7234	10.89%	12.44%
data 4-99c	0.6099	0.7001	0.7337	16.87%	4.58%
data 5-99c	0.6697	0.7025	0.7762	13.72%	9.49%
data 6-99c	0.4150	0.6172	0.7888	47.39%	21.75%

To verify the statistical significance of the observed performance differences, we conduct the Wilcoxon signed-rank test to compare the HV values of PPO-MOEA/D against MOEA/D and NSGA-II on each instance. The significance level is set at α=0.05, and each algorithm is independently run 30 times per instance. The test results are presented in Table 13, where “+” indicates that PPO-MOEA/D is significantly better than the comparison algorithm, “−” indicates significantly worse, and “≈” indicates no significant difference; p-values are shown in parentheses.

**Table 13:** Statistical significance test results (Wilcoxon signed-rank test).

Instance	PPO-MOEA/D vs MOEA/D	PPO-MOEA/D vs NSGA-II
Level 1-scale		
data 1-9c	+ (0.008)	+ (0.014)
data 2-9c	≈ (0.064)	≈ (0.071)
data 3-9c	+ (0.021)	+ (0.028)
data 4-9c	− (0.036)	≈ (0.085)
data 5-9c	+ (0.006)	+ (0.012)
data 6-9c	+ (0.009)	+ (0.008)
Level 2-scale		
data 1-49c	+ (0.022)	+ (0.018)
data 2-49c	+ (0.010)	≈ (0.052)
data 3-49c	+ (0.017)	+ (0.026)
data 4-49c	+ (0.031)	+ (0.019)
data 5-49c	+ (0.024)	+ (0.012)
data 6-49c	+ (0.007)	+ (0.015)
Level 3-scale		
data 1-74c	≈ (0.068)	+ (0.021)
data 2-74c	+ (0.023)	+ (0.017)
data 3-74c	+ (0.005)	+ (0.004)
data 4-74c	+ (0.012)	+ (0.008)
data 5-74c	≈ (0.059)	≈ (0.066)
data 6-74c	+ (0.008)	+ (0.006)
Level 4-scale		
data 1-99c	+ (0.018)	+ (0.014)
data 2-99c	+ (0.006)	+ (0.008)
data 3-99c	+ (0.025)	+ (0.016)
data 4-99c	+ (0.014)	+ (0.027)
data 5-99c	+ (0.019)	+ (0.022)
data 6-99c	+ (0.005)	+ (0.006)

Among all 24 test instances, PPO-MOEA/D is significantly better than MOEA/D in 18 instances (+), shows no significant difference in 4 instances (≈), and is significantly worse in 2 instances (−), achieving a win rate of 75.0%. Compared with NSGA-II, PPO-MOEA/D is significantly better in 19 instances (+), shows no significant difference in 4 instances (≈), and is significantly worse in 1 instance (−), achieving a win rate of 79.2%.

On small-scale (10-node) instances, PPO-MOEA/D is significantly better than MOEA/D in 3 instances, shows no significant difference in 2, and is significantly worse in 1; compared with NSGA-II, it is significantly better in 3 instances, shows no significant difference in 2, and is significantly worse in 1. This indicates that the performance of the three algorithms is relatively close on small-scale problems, and the advantages of PPO-MOEA/D are not yet prominent. On large-scale (75-node and 100-node) instances, PPO-MOEA/D is significantly better than MOEA/D in 10 instances, shows no significant difference in 1, and is significantly worse in 1; compared with NSGA-II, it is significantly better in 10 instances and shows no significant difference in 2. This reflects that as the problem scale increases, the performance advantages of PPO-MOEA/D become more pronounced and stable.

These statistical results demonstrate that the performance advantages of PPO-MOEA/D over the two benchmark algorithms are statistically significant on the majority of instances, and are not attributable to stochastic fluctuations of the algorithms. Meanwhile, the instances with no significant difference or even slightly worse performance also indicate that the improvement effect of PPO-MOEA/D is closely related to problem scale, on small-scale simple search spaces, the training benefits of the reinforcement learning strategy are limited and its advantages are less evident; on large-scale complex search spaces, the dynamic initialization and adaptive operator selection mechanisms of PPO-MOEA/D can play a greater role.

### Analysis of objective function values considering the decision of vehicle departure times at nodes

To investigate the impact of the decision regarding vehicle departure times at each node on transportation cost and transportation risk, two scenarios are considered for calculating the objective function values. The first scenario sets the departure time of all vehicles to 0, with vehicles not waiting at any customer nodes throughout the route. The second scenario allows vehicles to decide whether to wait at customer nodes. Experiments are conducted on instances with 10, 50, 75, and 100 customer nodes, and the resulting solutions are shown in Table 14.

**Table 14:** Comparison of objective function values considering the decision of vehicle departure times at nodes.

Scale	Strategies	Average Total Transportation Risk	Average Total Transportation Cost	Relative Change Rate of Risk	Relative Change Rate of Cost
Level 1-scale	Waiting	28.1 ± 6.3	394.4 ± 6.2	−32.3%	+2.9%
	Non-Waiting	41.5 ± 5.7	383.2 ± 9.9	–	–
Level 2-scale	Waiting	77.2 ± 18.5	3978.7 ± 34.5	−37.1%	+0.6%
	Non-Waiting	122.8 ± 27.2	3954.2 ± 42.1	–	–
Level 3-scale	Waiting	95.7 ± 12.7	5420.8 ± 69.6	−46.1%	−5.7%
	Non-Waiting	177.4 ± 14.5	5745.3 ± 65.4	–	–
Level 4-scale	Waiting	106.5 ± 18.4	6985.7 ± 96.3	−44.9%	−1.2%
	Non-Waiting	193.1 ± 22.8	7068.4 ± 87.5	–	–

In the four scales of instances, the introduction of the waiting strategy significantly reduced the total transportation risk, with reductions ranging from 32% to 46%. Notably, the Level 3-scale and Level 4-scale instances exhibited larger reductions, indicating that the optimization effect brought by the waiting strategy is more pronounced in scenarios with larger node scales. This suggests that, in a time-varying risk environment, reasonable waiting to avoid high-risk periods can effectively enhance transportation safety.

Regarding the change in total transportation cost, the trend shows an initial increase followed by a decrease. In the Level 1-scale and Level 2-scale instances, the waiting strategy caused a slight increase in cost, rising by 2.9% and 0.6%, respectively. Since these two scales involve fewer nodes, the time windows available for optimization are narrower. If the waiting time is too long, it generates penalty costs that offset, or even surpass, the savings from avoiding high-risk periods. Therefore, despite the risk reduction, the total cost slightly increased.

In the Level 3-scale and Level 4-scale instances, with a larger node scale and more flexible vehicle scheduling and time windows, the waiting strategy can better coordinate vehicle departure times and optimize total transportation costs. The overall benefits of the waiting strategy outweigh the potential penalty costs, thus reducing both the total transportation risk and the total transportation cost.

### Sensitivity analysis

In the hazardous materials transportation route optimization problem based on time-varying road networks, the optimal solution of the model is frequently affected by various pivotal parameters, such as fluctuations in population size, risk values, and other factors. Given that these parameters exhibit inherent uncertainty, variability, and fluctuations in real-world scenarios, conducting a sensitivity analysis is imperative to evaluate the model’s stability and adaptability across different parameter ranges. Such an analysis aids in identifying key influencing factors and provides valuable insights for the development of more resilient transportation strategies. This section introduces two types of sensitivity analysis experiments: variations in population size and alterations in time-period risk. These experiments are executed on instances with 10, 50, 75, and 100 customer nodes.

#### Sensitivity analysis considering population size variation.

Population size constitutes a pivotal parameter that profoundly influences the performance and computational efficiency of multi-objective evolutionary algorithms. A population that is overly small may suffer from insufficient diversity, thereby causing the algorithm to stagnate in local optima, while a population that is excessively large can result in a substantial increase in the computational cost per generation, consequently impeding the convergence rate. To ascertain the optimal population size for the PPO-MOEA/D algorithm presented in this study, a series of experiments were conducted on instances featuring 10, 50, 75, and 100 customer nodes. Three distinct population sizes 300, 500, and 700 were systematically evaluated to gauge the algorithm’s performance across these scenarios, with all other algorithmic parameters held constant. For each specific “problem size-population size” pairing, the algorithm’s efficacy was assessed through the metrics of average solution time and hypervolume (HV). The results, detailed in Table 15, present the average values and standard deviations for each data point, derived from six different instances of the corresponding scale, each executed 10 times.

**Table 15:** Performance comparison of different population sizes.

Scale	Population size	Computation time (s)	HV
Level 1-scale	300	16 ± 3.2	0.671 ± 0.012
	500	33 ± 2.8	0.864 ± 0.009
	700	54 ± 5.4	0.866 ± 0.007
Level 2-scale	300	56 ± 4.5	0.750 ± 0.014
	500	68 ± 5.7	0.833 ± 0.011
	700	129 ± 7.7	0.836 ± 0.010
Level 3-scale	300	81 ± 2.4	0.722 ± 0.015
	500	102 ± 5.8	0.857 ± 0.013
	700	186 ± 5.2	0.860 ± 0.012
Level 4-scale	300	101 ± 3.9	0.696 ± 0.020
	500	124 ± 4.6	0.843 ± 0.016
	700	247 ± 7.2	0.848 ± 0.015

The solution time shows a high sensitivity to the increase in population size. Larger populations typically require more iterations to achieve stable convergence, which further increases the time cost. For the Level 1-scale instances, when the population size is 500, the solution time increases by 63.6% compared to the population size of 300. When the population size is increased from 500 to 700, the solution time for a population size of 700 is significantly higher than that for a population size of 500. This effect is especially pronounced in the Level 3-scale and Level 4-scale instances, where the solution time for a population size of 700 is almost double that for a population size of 500.

The HV metric exhibits a typical diminishing marginal returns pattern as the population size increases. For example, in the Level 3-scale instances, when the population size increases from 300 to 500, the mean HV rises from 0.722 to 0.857, a relative improvement of 18.7%, demonstrating significant performance enhancement. However, when the population size is further increased from 500 to 700, the mean HV only increases slightly from 0.857 to 0.860, with a relative improvement of just 0.35%. Meanwhile, the solution time skyrockets from 102 seconds to 186 seconds, an increase of 82.4%. This means that to achieve a marginal quality improvement of just 0.35%, nearly 82.4% more computation time is required. This phenomenon is consistent across all problem sizes, with the HV improvement for population sizes from 500 to 700 not exceeding 0.5%.

Overall, a population size of 500 strikes a good balance between computational efficiency and solution quality. Both in terms of solution time and HV value, a population size of 500 generally demonstrates the best performance across most scales. While increasing the population size can improve the HV value in certain cases, the magnitude of this improvement may not be sufficient to offset the substantial increase in computation time, particularly for larger problem sizes.

#### Sensitivity analysis considering changes in risk for each time period.

To investigate the impact of time-varying risks in hazardous materials transportation on the objective function, the risk for each of the 24 time periods is increased and decreased by 20%. Experiments are conducted on instances with 10, 50, 75, and 100 customer nodes. The resulting Pareto fronts are shown in Figs 14–17 and the hypervolume metric values are provided in Table 16.

**Table 16:** Hypervolume comparison under changes in risk for each time period.

Group	$r_{ij}^{p}$	$r_{ij}^{p}+20\%$	Change rate	$r_{ij}^{p}-20\%$	Change rate
data 1-9c	0.8042	0.7764	−3.46%	0.8572	+6.69%
data 2-9c	0.8766	0.8631	−1.54%	0.9221	+5.19%
data 3-9c	0.8962	0.8642	−3.57%	0.9127	+1.84%
data 4-9c	0.8339	0.8165	−2.09%	0.8802	+5.55%
data 5-9c	0.7801	0.7618	−2.35%	0.8258	+5.86%
data 6-9c	0.8209	0.7845	−4.43%	0.8744	+6.52%
data 1-49c	0.7785	0.7425	−4.62%	0.7344	−5.66%
data 2-49c	0.6933	0.6714	−3.16%	0.7276	+4.95%
data 3-49c	0.8390	0.8082	−3.67%	0.8818	+5.10%
data 4-49c	0.8148	0.7723	−5.22%	0.8663	+6.32%
data 5-49c	0.8275	0.8035	−2.90%	0.8891	+7.44%
data 6-49c	0.7313	0.7167	−2.00%	0.7911	+8.18%
data 1-74c	0.8576	0.8189	−4.51%	0.8925	+4.07%
data 2-74c	0.8243	0.7736	−6.15%	0.9124	−0.23%
data 3-74c	0.8034	0.7980	−0.67%	0.8723	+8.58%
data 4-74c	0.8136	0.7860	−3.39%	0.8977	+10.34%
data 5-74c	0.8721	0.8359	−4.15%	0.8849	+1.47%
data 6-74c	0.7219	0.6752	−6.47%	0.8106	+12.29%
data 1-99c	0.8603	0.7051	−18.04%	0.9319	+8.32%
data 2-99c	0.8243	0.7239	−12.18%	0.8952	+8.60%
data 3-99c	0.8214	0.7677	−6.54%	0.9033	+9.97%
data 4-99c	0.8337	0.7680	−7.88%	0.8203	−1.61%
data 5-99c	0.8125	0.7519	−7.46%	0.8938	+10.01%
data 6-99c	0.7743	0.7027	−9.25%	0.8611	+11.21%

**Fig 14:**
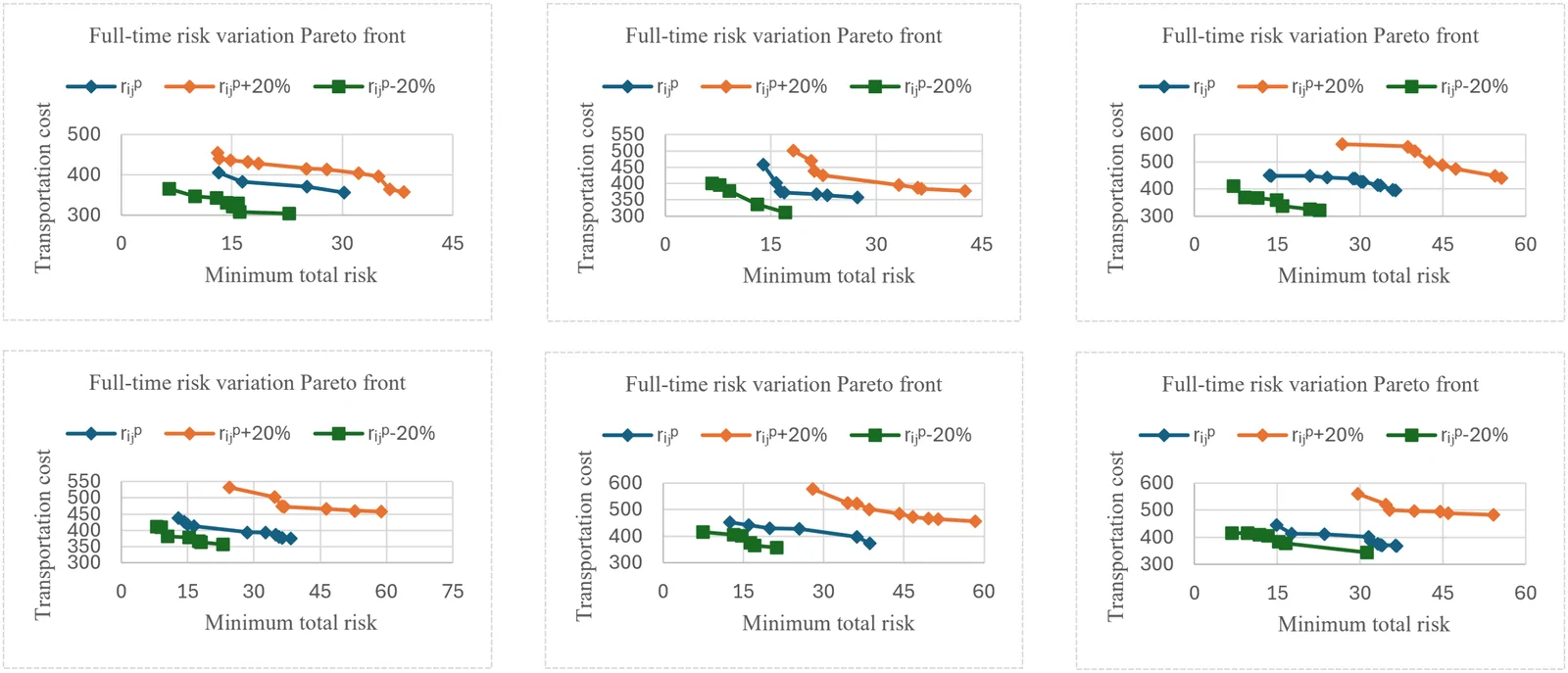
Pareto fronts for the 10-node instance under changes in risk for each time period.

**Fig 15:**
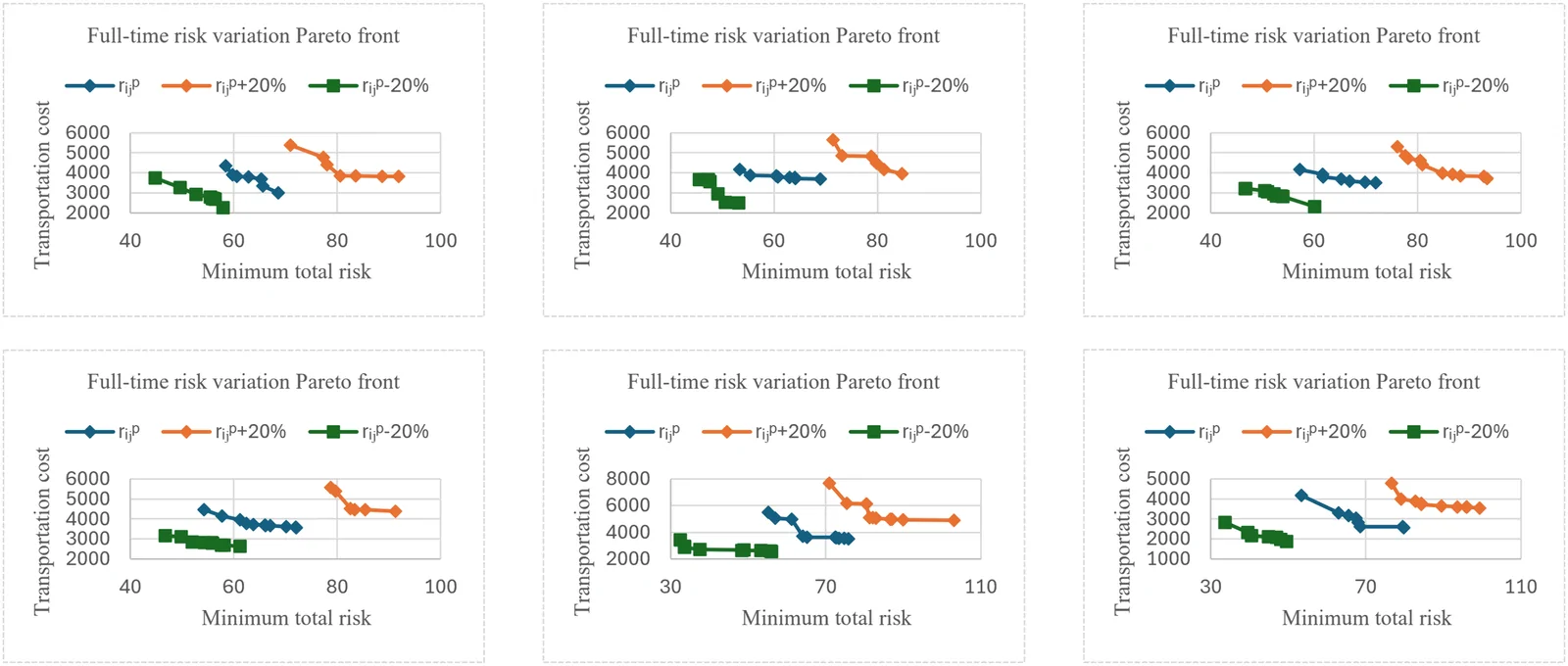
Pareto fronts for the 50-node instance under changes in risk for each time period.

**Fig 16:**
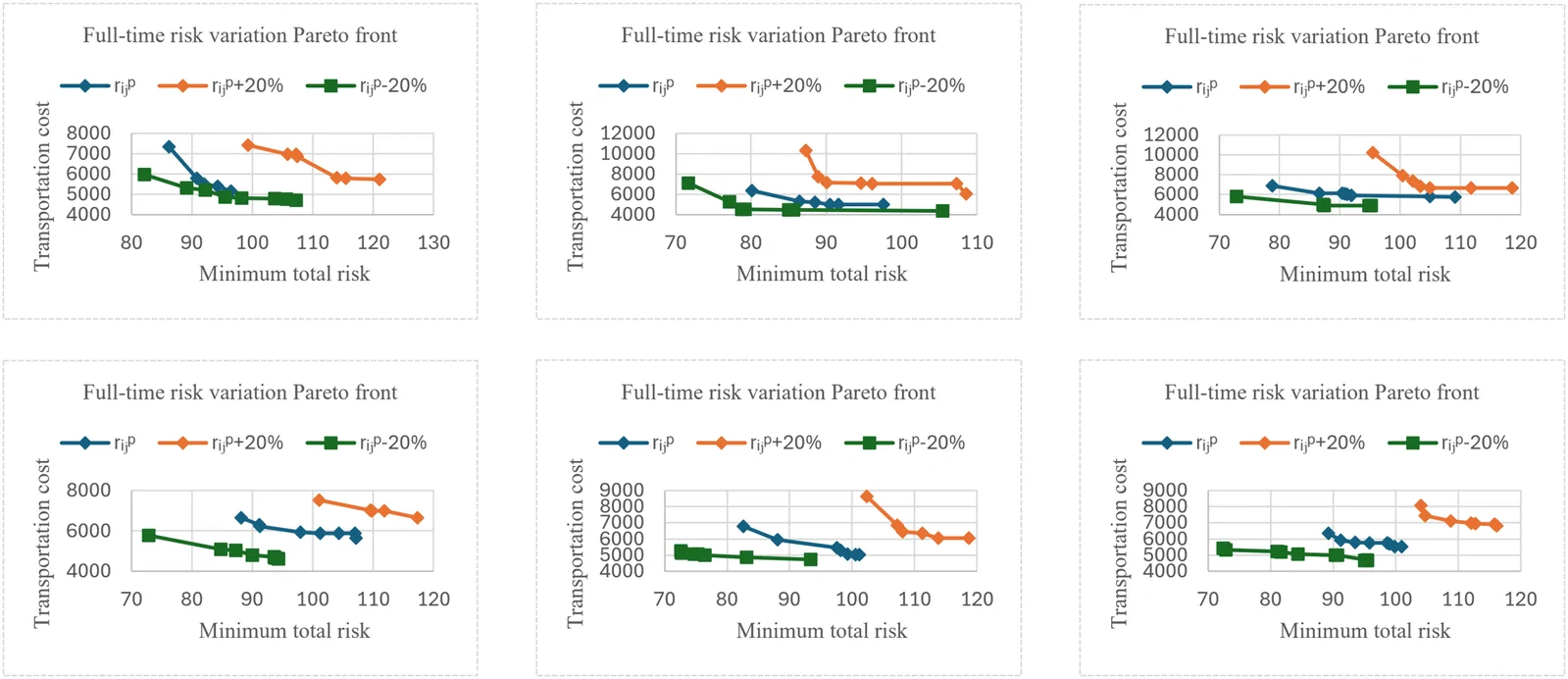
Pareto fronts for the 75-node instance under changes in risk for each time period.

**Fig 17:**
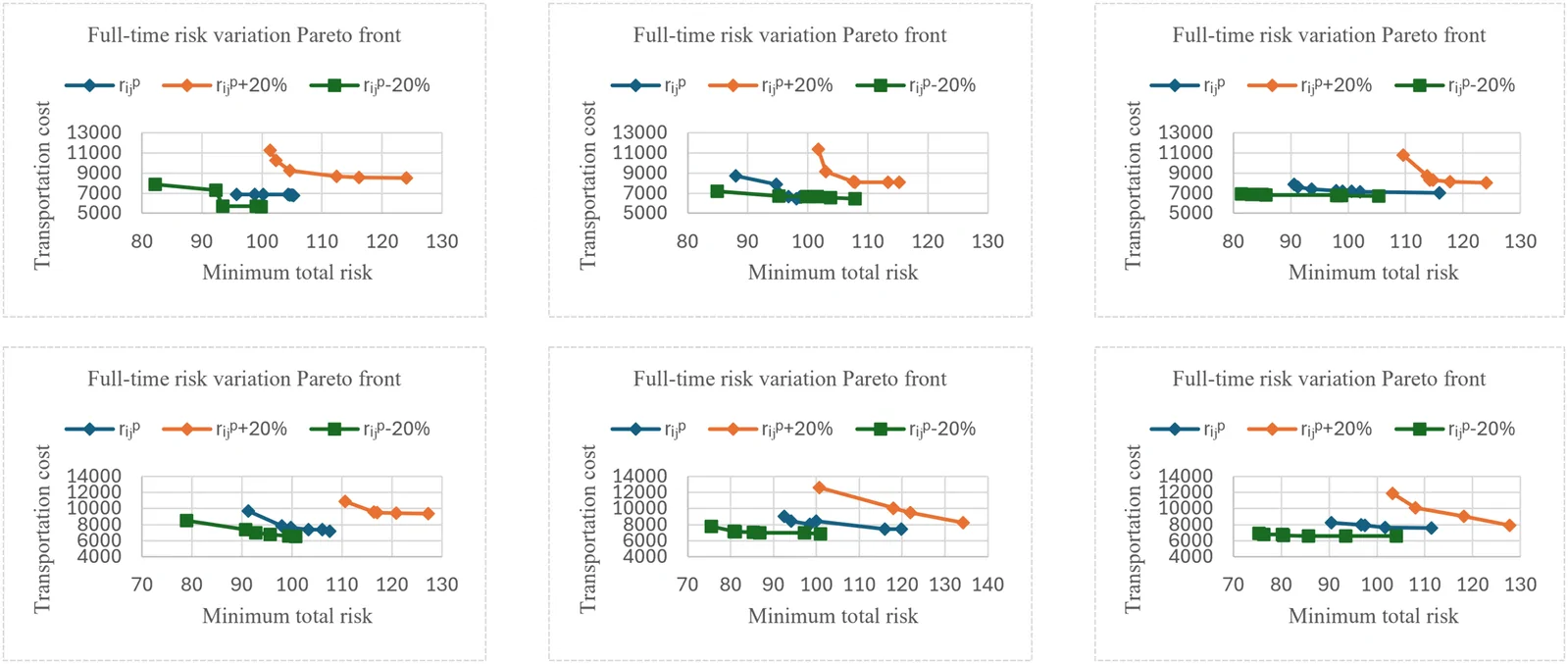
Pareto fronts for the 100-node instance under changes in risk for each time period.

The experimental results show that fluctuations of ±20% in the risk for each time period have a significant impact on the time-varying hazardous materials transportation scheduling. By comparing the changes in the Pareto front shapes and hypervolume metrics across four node scales (10, 50, 75, 100), it is found that the variation in risk for each time period not only directly alters the quality of the solution set but also highlights the effectiveness and scale sensitivity of the waiting strategy.

When the risk value across all time periods of the transportation network is systematically increased by 20%, the Pareto front deteriorates across all four scale levels. Numerically, the average hypervolume for the 10-node instance decreases by 5.0%, while the HV reduction for the 100-node system reaches 11.45%, demonstrating a clear scale effect. This deterioration is manifested as a shift of the entire Pareto front towards a “high-risk-high-cost” region. This indicates that when every time period is under high risk, the trade-off space in multi-objective optimization is squeezed. The effectiveness of the waiting strategy is weakened in this scenario because the risk increases across all time periods, and simply delaying departure times cannot identify low-risk windows.

In contrast to the increase in risk across all time periods, a 20% decrease in the risk value across all time periods has a beneficial impact on overall optimization. The HV metrics for systems of various scales generally improve by 5%−13%, and the Pareto front significantly expands towards the lower-left corner, indicating a lower risk level achieved under the given cost constraints. The overall reduction in risk unleashes the potential of the waiting strategy, leading to improvements in both risk and cost.

By comparing the response differences across the four scale instances, it is observed that the system scale is positively correlated with sensitivity to risk fluctuations. For the 10-node instance, the HV variation under a 20% risk fluctuation ranges from 1.54% to 4.43%, while for the 100-node instance, the variation increases to nearly 5% to 18%. This scale effect is reflected in the change of the Pareto front shape, where the “shrinkage-expansion” magnitude of the front for larger-scale systems is much greater than that of smaller-scale systems. The reason for this is that larger-scale networks require more vehicles, and the paths become more complex, with global risk changes being amplified through network effects.

In summary, the impact of risk fluctuations across each time period on the waiting strategy demonstrates a systematic dual effect of compression and release, which intensifies as the scale increases. The study results show that a 20% increase in risk will reduce the optimization space by nearly 5.24%, while an equivalent decrease in risk can provide an optimization gain of approximately 5.88%. This finding provides a basis for dynamic decision-making: during periods of increased risk, the waiting window should be shortened to control risk exposure, whereas during periods of decreased risk, waiting times can be extended to enhance optimization potential.

#### Sensitivity analysis considering changes in risk for selected time periods.

To compare the impact of risk fluctuations in each time period on time-varying hazardous materials transportation, this section considers the random selection of 12 out of the 24 time periods. The risk for these selected periods is increased and decreased by 20%. Experiments are conducted on instances with 10, 50, 75, and 100 customer nodes. The resulting Pareto fronts are shown in Figs 18–21 and the hypervolume metrics are provided in Table 17.

**Table 17:** Hypervolume comparison under changes in risk for selected time periods.

Group	$r_{ij}^{p}$	$r_{ij}^{p}+20\%$	Change rate	$r_{ij}^{p}-20\%$	Change rate
data 1-9c	0.8212	0.8064	−1.80%	0.8356	+1.75%
data 2-9c	0.8567	0.8431	−1.59%	0.8713	+1.70%
data 3-9c	0.9053	0.8842	−2.33%	0.9347	+3.25%
data 4-9c	0.8688	0.8365	−3.72%	0.8901	+2.45%
data 5-9c	0.8615	0.8518	−1.13%	0.8770	+1.80%
data 6-9c	0.8797	0.8545	−2.86%	0.8934	+1.56%
data 1-49c	0.7985	0.7851	−1.68%	0.8251	+3.33%
data 2-49c	0.8558	0.8422	−1.59%	0.8710	+1.78%
data 3-49c	0.8408	0.8328	−0.95%	0.8624	+2.57%
data 4-49c	0.8148	0.8023	−1.53%	0.8342	+2.38%
data 5-49c	0.8627	0.8529	−1.14%	0.8316	−3.60%
data 6-49c	0.8314	0.8309	−0.06%	0.8518	+2.45%
data 1-74c	0.8612	0.8546	−0.77%	0.8895	+3.29%
data 2-74c	0.8238	0.8046	−2.33%	0.8489	+3.05%
data 3-74c	0.8220	0.8018	−2.46%	0.8593	+4.54%
data 4-74c	0.8009	0.7875	−1.67%	0.8325	+3.95%
data 5-74c	0.8090	0.8011	−0.98%	0.8233	+1.77%
data 6-74c	0.7488	0.7314	−2.32%	0.7611	+1.64%
data 1-99c	0.8320	0.8057	−3.16%	0.8419	+1.19%
data 2-99c	0.8321	0.8156	−1.98%	0.8603	+3.39%
data 3-99c	0.8023	0.7899	−1.55%	0.7926	−1.21%
data 4-99c	0.8374	0.8212	−1.93%	0.8631	+3.07%
data 5-99c	0.8045	0.7847	−2.46%	0.8335	+3.60%
data 6-99c	0.7955	0.7792	−2.05%	0.8358	+5.07%

**Fig 18:**
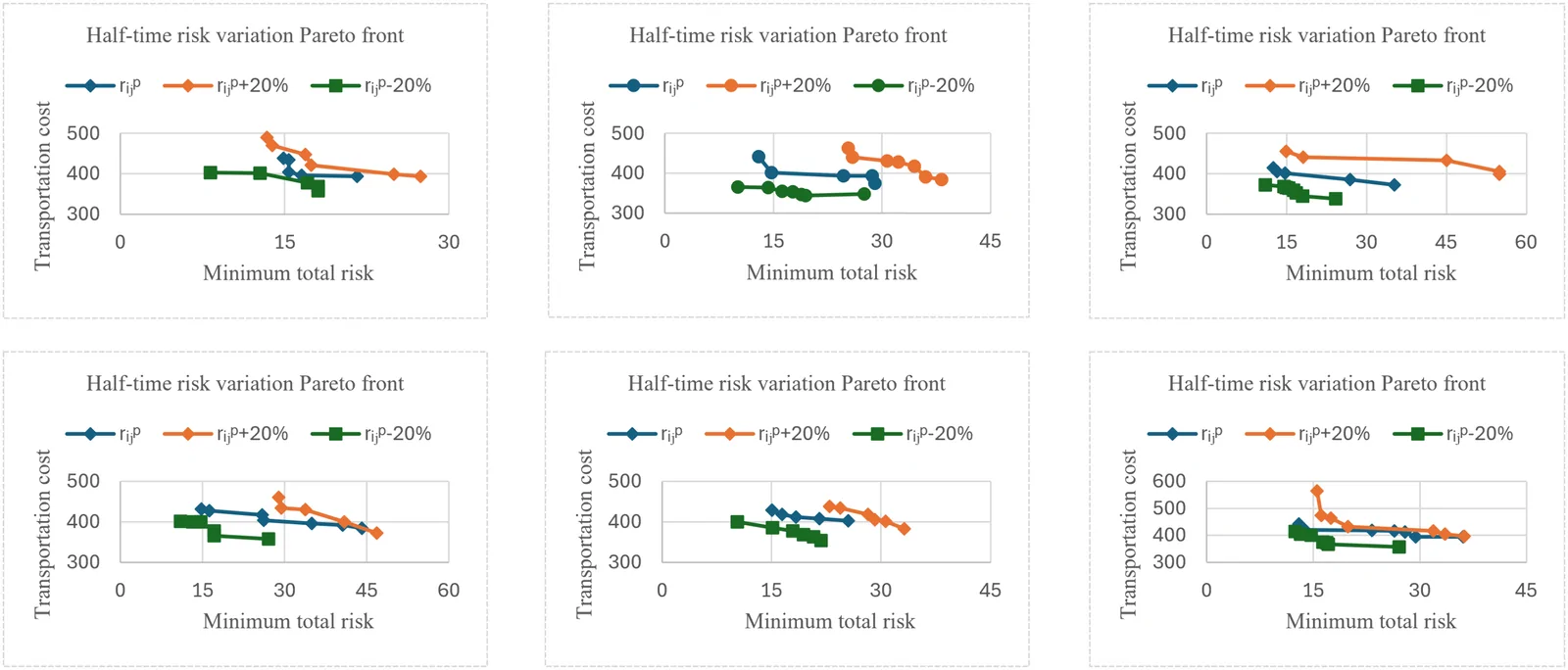
Pareto fronts for the 10-node instance under changes in risk for selected time periods.

**Fig 19:**
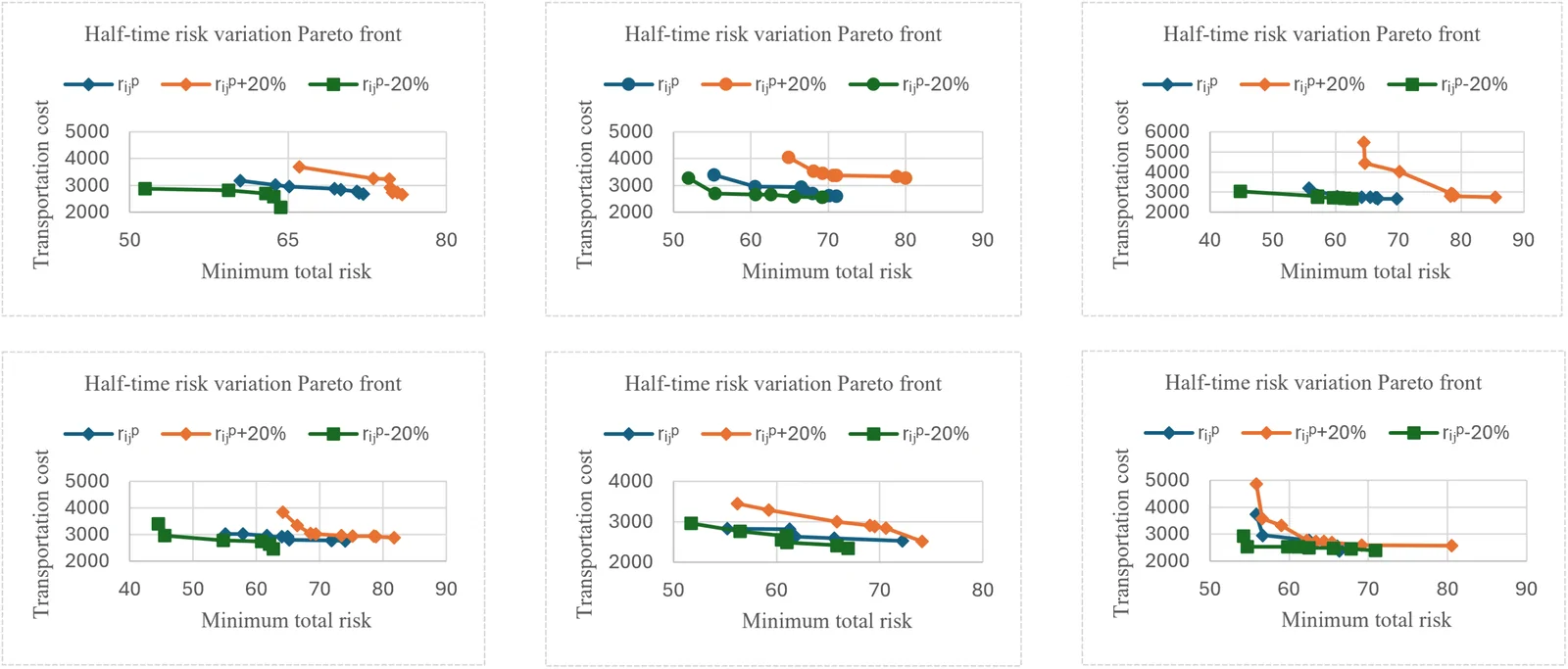
Pareto fronts for the 50-node instance under changes in risk for selected time periods.

**Fig 20:**
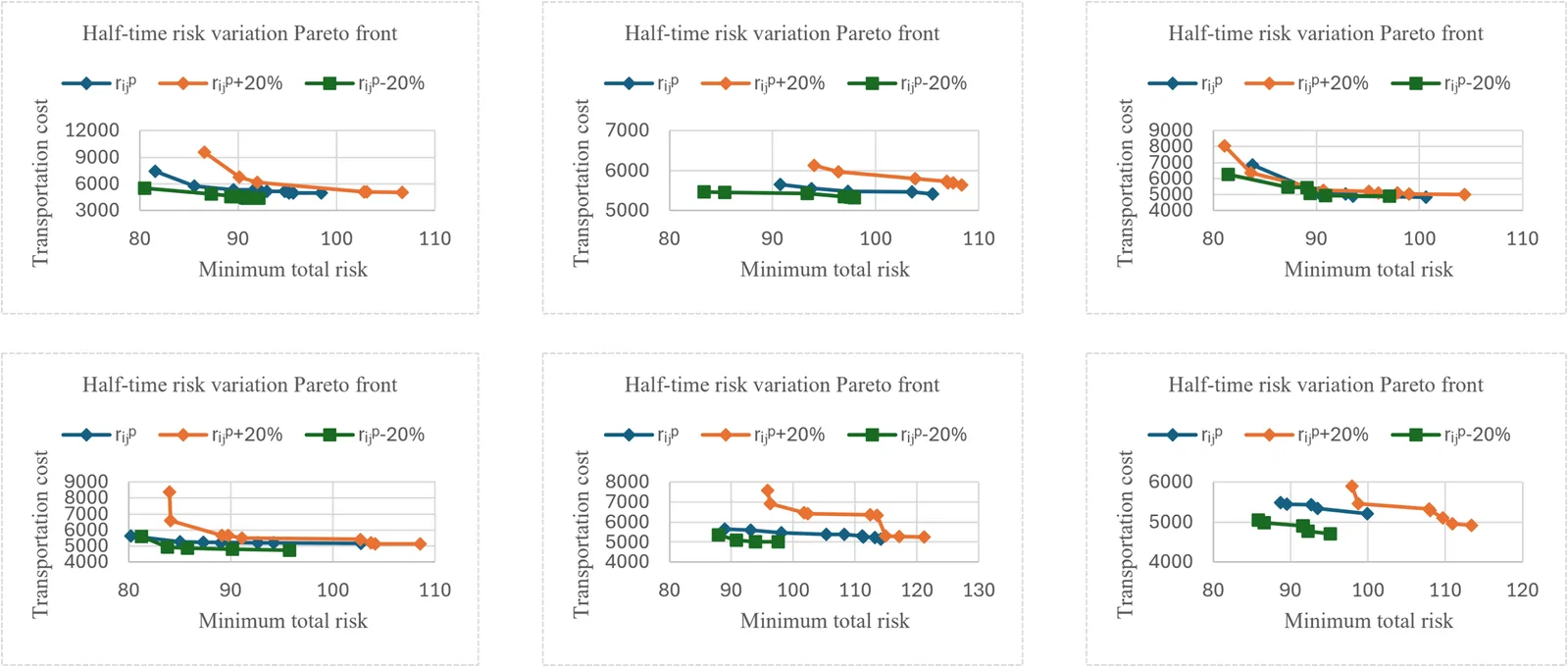
Pareto fronts for the 75-node instance under changes in risk for selected time periods.

**Fig 21:**
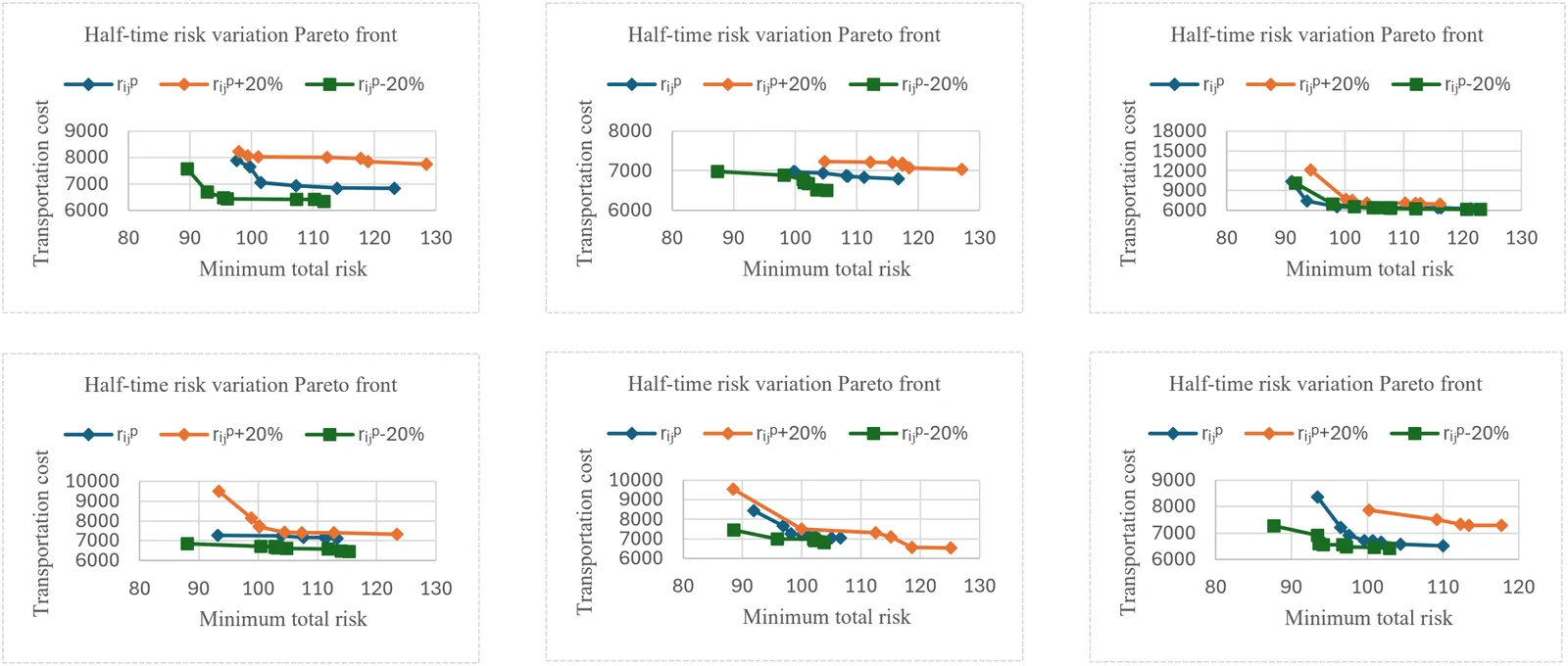
Pareto fronts for the 100-node instance under changes in risk for selected time periods.

Compared to the risk fluctuations across all time periods, the local changes in risk (in 12 randomly selected time periods) by an increase or decrease of 20% show more complex spatiotemporal heterogeneity in their impact on the waiting strategy. By analyzing the Pareto fronts and hypervolume metrics of the instances across four node scales, it is found that the overall impact of random changes in risk for selected time periods is smaller than that of the risk fluctuations in every time period. However, such changes lead to a differentiation in the solution set structure, thereby providing a specific pathway for the selective optimization of waiting strategies based on time periods.

The overall impact of a 20% increase or decrease in risk for randomly selected time periods on the optimization space is significantly smaller than that of risk fluctuations across all time periods. Numerical results show that in the scenario where risk increases by 20%, the HV decrease ranges from 1.0% to 3.0%, which is only 20% of the decrease seen in scenarios where risk increases across all time periods. Particularly, for the 10-node system, a 20% increase in risk for selected time periods leads to a 2.24% decrease in HV, while a 20% increase in risk for each time period results in a 2.9% decrease, indicating that the impact of local changes is weaker. This change in impact is due to the locality of the risk fluctuations, where only half of the time periods experience changes in risk values. The system can still adjust vehicle departure times to avoid risk fluctuation periods. When the risk for selected time periods decreases by 20%, the HV increase, ranging from 1.0% to 5.0%, is also smaller than the increase observed in scenarios where risk decreases across all time periods. This indicates that when facing fluctuations in risk across selected time periods, the system can adjust the departure times of certain vehicles to respond. However, this adjustment capability is constrained by the overall scheduling complexity. For example, when the risk in some time periods decreases, although more vehicles can be scheduled to depart during these “favorable periods,” if the system scale is too large and the paths are too complex, it becomes difficult to coordinate all vehicles to these periods, causing some optimization opportunities to be missed. From this, it can be concluded that when encountering risk fluctuations in only certain time periods, there is no need to adjust the entire scheduling strategy. A minor adjustment of the departure times for vehicles in the affected time periods is sufficient to effectively control the risk.

A significant characteristic of risk fluctuations in selected time periods is the apparent “differentiation phenomenon” in the Pareto front. In scenarios where the risk for selected time periods increases by 20%, the transportation solutions derived for the four scales are clearly divided into two groups, corresponding to solution sets with different time period selection strategies: one representing the strategy of “taking risks and departing during high-risk periods” and the other representing the strategy of “delaying departure until risk decreases.” Taking the 75-node instance as an example, at intermediate risk levels, the distribution of solutions becomes sparse. This differentiation reflects the system’s two response strategies when facing localized high-risk periods: one is to accept the risk and depart on time to control costs, and the other is to delay departure to a low-risk period, potentially increasing waiting costs. This structural differentiation provides decision-makers with distinct strategy options, rather than merely passively accepting a deterioration in the overall solution when risk fluctuates in every time period.

By comparing the response of different scale instances to partial risk fluctuations, it is observed that the 50-node and 75-node instances exhibit better adaptability. The data show that for the 50-node instance, the HV decrease under a 20% risk increase in selected time periods is 1.16%, while the system-wide decrease for the 100-node instance reaches 2.19%. This highlights the trade-off between system complexity and robustness: the 10-node instance has a simple scheduling process and is minimally affected, while the 100-node instance, due to its scheduling complexity, amplifies local disturbances. In contrast, the 50-node and 75-node instances strike an optimal balance between complexity and flexibility, effectively mitigating local fluctuations through moderate path redistribution and time period adjustments, resulting in more stable overall performance. This finding has important implications for network scheduling, suggesting that decomposing a large-scale transportation network into moderately sized sub-networks may enhance the system’s resistance to local disturbances when facing localized risks.

In summary, the impact of partial risk fluctuations on the waiting strategy exhibits a composite set of characteristics: limited influence, structural differentiation, time period variation, and scale sensitivity. The study shows that the loss of optimization space due to a 20% increase in partial period risk is significantly lower than that of global fluctuations, but it triggers a strategic differentiation in the Pareto front, creating different solution sets based on time period selection. This differentiation indicates that by identifying and prioritizing the avoidance of specific high-risk periods, risk control can be achieved without significantly altering overall performance. From this, a management insight can be drawn: for low-risk fluctuations, the current waiting strategy can be maintained; for moderate-risk fluctuations, selective waiting optimization based on time periods can be considered; and for high-risk fluctuations, a comprehensive adjustment involving path redistribution is necessary. Notably, medium-scale transportation networks exhibit the best adaptability balance, providing a foundation for the scale optimization design of time-varying hazardous materials transportation networks that consider waiting strategies.

## Conclusion

This paper addresses the urban hazardous materials transportation vehicle routing optimization problem within time-varying road networks. The decision variables encompass both the vehicle’s travel route and the departure time at each node along the route, with the objective of minimizing the total transportation risk and overall transportation cost, which includes travel expenses and overtime penalty costs.

In light of the unique characteristics of urban hazardous materials transportation route optimization under time-varying road networks, an approximate algorithm, PPO-MOEA/D, is proposed using reinforcement learning to address the problem. Unlike the conventional MOEA/D algorithm, this approach integrates three key improvements to boost its performance. Firstly, to mitigate computational time and complexity, a preprocessing step is introduced for optimizing node departure time decisions at non-critical nodes. Secondly, reinforcement learning (PPO) is employed to train an agent capable of dynamically generating high-quality initial solutions for the MOEA/D algorithm. Lastly, the PPO-trained agent actively selects crossover and mutation operators tailored to the specific characteristics of each iteration phase, thereby enhancing the evolutionary efficiency of MOEA/D.

The proposed model and algorithm are validated through numerical experiments, with results demonstrating that optimizing node departure times significantly reduces transportation risk. In terms of algorithm performance, PPO-MOEA/D outperforms the conventional MOEA/D in convergence speed, solution quality, and computational efficiency. The effects of varying population sizes on computational efficiency and solution quality, as well as the variations in total transportation risk across different time periods, are analyzed. First, the node waiting strategy leads to reductions in both total transportation risk and total transportation cost. Second, the impact of risk fluctuations during each time period on the waiting strategy reveals a systematic compression and release effect, which intensifies as the scale increases. Third, the influence of partial risk fluctuations on the waiting strategy displays a complex range of characteristics, including limited impact, structural differentiation, time-period variation, and scale sensitivity. These findings provide valuable insights for hazardous materials transportation planning in synthetic network settings, and suggest potential directions for further validation on real-world networks. Specifically, the results suggest that, under the synthetic test conditions, reducing the waiting window during high-risk periods and extending waiting times during low-risk periods may help mitigate risk exposure and enhance optimization potential. For low-risk fluctuations, maintaining the current waiting strategy appears effective; for moderate-risk fluctuations, selective waiting optimizations based on time periods may be worth considering; and for high-risk fluctuations, comprehensive adjustments involving path redistribution may be necessary. These managerial observations, however, are derived from synthetic networks and require careful calibration and validation before being applied to real-world operations.

Future research can be developed in two directions. First, future studies should place more emphasis on the integration of multi-dimensional risk perception, considering the impact of different types of risks on the transportation process and incorporating these risks into the decision framework for waiting strategies. Specifically, multi-dimensional factors such as environmental conditions, accident probabilities, and population density could be integrated. During high-risk periods, reasonable node waiting could be employed to reduce potential accident risks, and based on this, coordination between waiting times and transportation routes could be optimized to reduce total risk. Second, future research could further combine dynamic traffic and time-varying risks to establish a multi-level decision model. This model would not only focus on individual decisions (such as vehicle routes and waiting timings) but would also dynamically adjust paths and waiting strategies based on real-time traffic and risk information through a feedback mechanism. Additionally, the application of the proposed model and algorithm to real-world network data, such as actual urban road networks with empirically derived risk and demand parameters, constitutes a necessary next step to assess their practical utility and to complement the synthetic-network-based findings of this study.

## Supporting information

S1 Data Synthetic test instances for VRP experiments. This file contains all generated test instances used in the computational experiments. The data are provided in standard format for easy access and reproduction.
(ZIP)
